# Crystal structures of APOBEC3G N-domain alone and its complex with DNA

**DOI:** 10.1038/ncomms12193

**Published:** 2016-08-02

**Authors:** Xiao Xiao, Shu-Xing Li, Hanjing Yang, Xiaojiang S. Chen

**Affiliations:** 1 Genetic, Molecular and Cellular Biology Program, Keck School of Medicine, University of Southern California, Los Angeles, California 90089, USA; 2 Molecular and Computational Biology Program, Departments of Biological Sciences and Chemistry, University of Southern California, Los Angeles, California 90089, USA; 3 Center of Excellence in NanoBiophysics, University of Southern California, Los Angeles, California 90089, USA; 4 Norris Comprehensive Cancer Center, University of Southern California, Los Angeles, California 90089, USA

## Abstract

APOBEC3G (A3G) is a potent restriction factor of HIV-1. The N-terminal domain of A3G (A3G-CD1) is responsible for oligomerization and nucleic acid binding, both of which are essential for anti-HIV activity. As a countermeasure, HIV-1 viral infectivity factor (Vif) binds A3G-CD1 to mediate A3G degradation. The structural basis for the functions of A3G-CD1 remains elusive. Here, we report the crystal structures of a primate A3G-CD1 (rA3G-CD1) alone and in complex with single-stranded DNA (ssDNA). rA3G-CD1 shares a conserved core structure with the previously determined catalytic APOBECs, but displays unique features for surface charge, dimerization and nucleic acid binding. Its co-crystal structure with ssDNA reveals how the conformations of loops and residues surrounding the Zn-coordinated centre (Zn-centre) change upon DNA binding. The dimerization interface of rA3G-CD1 is important for oligomerization, nucleic acid binding and Vif-mediated degradation. These findings elucidate the molecular basis of antiviral mechanism and HIV-Vif targeting of A3G.

## Abstract (web summary)

The N-terminal domain of APOBEC3G (A3G) is required for anti-HIV activity, HIV-1 viral infectivity factor (Vif) binds to this domain of A3G to mediate its degradation. Here, the authors use their crystal structures of this A3G domain alone and bound to single-stranded DNA to investigate the molecular details of this interaction.

## Graphical abstract

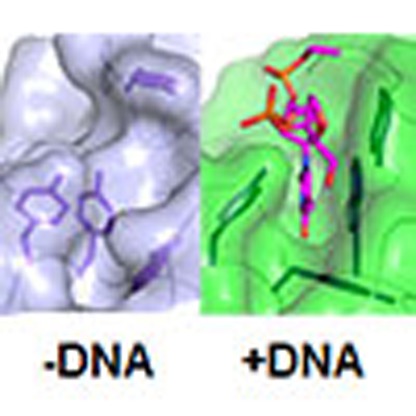

APOBEC3s (A3s) are deoxycytidine deaminases that have important roles in mammalian innate immune responses. Members of this family catalyze the conversion of cytosine into uracil on single-stranded DNA (ssDNA) and restrict retroviral replication and endogenous retroelements. All A3 enzymes contain a homologous cytidine deaminase domain (CD) defined by a conserved zinc-coordinating motif (H-X-E-X_23–28_-P-C-X_2–4_-C). In primates including human, the A3 family includes seven members consisting of either one (A3A, A3C, A3H) or two CDs (A3B, A3D, A3F, A3G), with the two domains following the naming convention of CD1 and CD2 when counted from the N to C terminus. According to sequence consensus, these domains can be further classified into Z1 (A3A, A3B-CD2, A3G-CD2), Z2 (A3B-CD1, A3C, A3D-CD1, A3D-CD2, A3F-CD1, A3F-CD2, A3G-CD1) and Z3 (A3H)^1^. Current studies have shown that single-domain A3s and CD2s of double-domain A3s are catalytically active, specifically targeting on TC or CC dinucleotide hotspot, whereas the CD1s are inactive and are proposed to be involved in protein oligomerization and nucleic acid binding^2^,^3^,^4^.

Among all the A3s, A3G is most known for being a potent restriction factor against HIV-1 (refs 5, 6). A3G has been shown to incorporate into budding virions and introduces massive deamination on the minus-strand viral ssDNA during reverse transcription^6^,^7^,^8^,^9^. The N-terminal CD1 is essential for virion incorporation, a process thought to be achieved through CD1–CD1 oligomerization and RNA binding^10^,^11^,^12^,^13^. The CD1 is also considered important for ssDNA substrate binding and can greatly enhance the deamination efficiency, processivity and directionality of the full-length A3G^14^,^15^. In addition, the CD1 domain has been shown to bind to viral RNA directly and hinder reverse transcription, as a potential deamination-independent antiviral mechanism^16^,^17^,^18^.

Nevertheless, the antiviral inhibition from A3G is counteracted by the HIV-1 protein Vif. Vif interacts with A3G-CD1 and recruits the components of the E3-ubiquitin ligase complex, including EloB, EloC, CBFβ and Cul5, leading to the degradation of A3G through the proteasomal pathway^19^,^20^,^21^,^22^,^23^,^24^. Although the detailed molecular interactions between A3G and Vif remain unclear, the most critical Vif-binding residues on A3G have been identified as DPD 128–130 between loop-7 and helix-4 (refs 25, 26, 27, 28, 29, 30, 31), among which the residue D128 governs the species-specific Vif sensitivity in primates^27^,^28^,^29^,^30^. In addition, other residues located on loop-1, loop-3 and loop-7 of an A3G-CD1 variant have been reported to interact with HIV-1 Vif^26^,^31^,^32^.

Structures of the catalytically active single-domain A3A and A3C, as well as the CD2 domains of A3B, A3F and A3G have been solved by X-ray crystallography or nuclear magnetic resonance (NMR)^15^,^33^,^34^,^35^,^36^,^37^,^38^,^39^,^40^,^41^. Although all N-terminal CD1 domains belong to the Z2 subfamily, their distinct biochemical properties and conserved consensus sequences suggest that CD1 is a unique subgroup different from the rest of the A3 domains (Fig. 1a). So far, structural information of any CD1 domain is limited, mostly due to the difficulties in obtaining the soluble CD1 protein. A recently reported NMR structure of a human A3G-CD1 variant (A3G-sNTD) overcame the solubility issue by mutating 20% of the residues, yielding a mutant variant that no longer oligomerizes, interacts with RNA, nor gets degraded by Vif^32^. Therefore, the high-resolution structure of a fully-functional CD1 is still unresolved.

Here, we report the high-resolution (2 Å) crystal structure of a nearly wild-type (WT) A3G-CD1 from rhesus macaque (*Macaca mulatta*) (rA3G-CD1). Unlike the NMR structure of A3G-sNTD, the rA3G-CD1 structure is in a dimeric form. The interactions of this dimerization interface are important for nucleic acids binding, CD1 oligomerization, HIV-1 Vif-binding and Vif-mediated degradation. We have also determined the co-crystal structure of rA3G-CD1 bound to a poly-dT ssDNA that reveals the conformational changes of the loops and residues around the Zn-centre induced by DNA binding. These findings elucidate the structural basis for oligomerization and nucleic acid binding by A3G and offer insight into A3G–Vif interactions, which are valuable for future anti-HIV therapeutic development.

## Results

### Soluble A3G-CD1 dimers after nucleic acid removal

**Figure 1:**
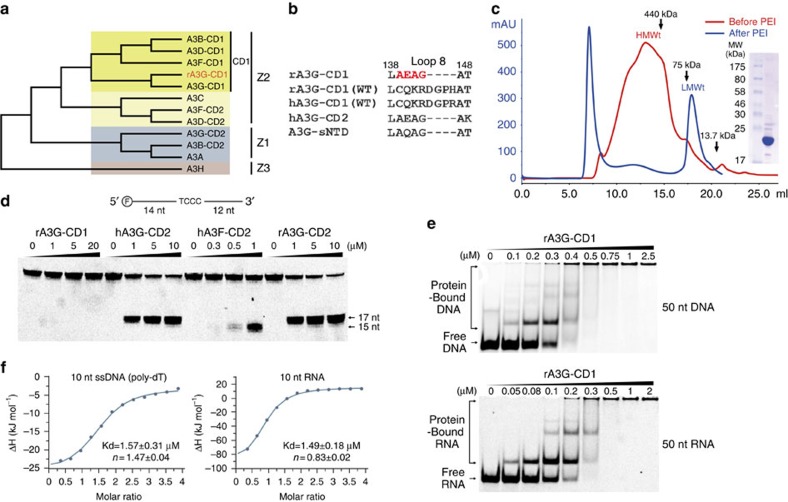
Purification and biochemical characterization of rA3G-CD1. (**a**) Phylogenetic tree of A3 domains, with rA3G-CD1 in red. (**b**) Sequence alignments of loop-8 region for rA3G-CD1 (used for crystallization), rA3G-CD1 (WT), hA3G-CD1 (WT), hA3G-CD2 and A3G-sNTD. The four residues in red are the only mutated sequence in the rA3G-CD1 construct. (**c**) Elution profile of rA3G-CD1 analyzed by size-exclusion chromatography (Suprose 6 10/300 GL) before (red) and after (blue) PEI treatment. Inset: SDS–PAGE shows the purified rA3G-CD1 from LMWt that was used for biochemical studies and crystallization. (**d**) Deamination assays of rA3G-CD1, hA3G-CD2, hA3F-CD2 and rA3G-CD2 with a 5′-FAM-labelled 30 nt ssDNA. 17 nt and 15 nt are the products by deamination of the 3rd and 1st C from 5′ end, respectively. (**e**) Electrophoresis mobility shift assays (EMSA) of purified rA3G-CD1 binding to 5′-FAM-labelled 50 nt ssDNA (top panel) and RNA (bottom panel). (**f**) ITC of rA3G-CD1 binding to short 10 nt ssDNA (poly-dT, left panel) and 10 nt RNA (right panel).

Since the CD1 of human A3G (hA3G) has poor solubility in *Escherichia coli*, we examined the expression and solubility of multiple A3G homologues from primates, and found that rA3G-CD1 is more soluble than hA3G-CD1. After a systematic mutational test on different regions throughout the CD1 sequence, we found that replacing loop-8 of rA3G-CD1 with four residues from hA3G-CD2 (CQKRDGPH→AEAG, Fig. 1b; Supplementary Figs 1,2b) further improved solubility. This rA3G-CD1 loop-8 mutant, referred to as rA3G-CD1 hereafter for simplicity, can be purified in large quantity. However, the purified rA3G-CD1 was initially found as high molecular weight (HMWt) aggregates that eluted in a broad peak from the Superose 6 size-exclusion chromatography (Fig. 1c). This peak fraction had a high A260/280 ratio (1.81), suggesting it has significant nucleic acid content. Fractions from the HMWt peak were subsequently treated with polyethylenimine (PEI), which is commonly used to remove DNA/RNA from the bound protein complex^42^. The HMWt aggregates were dissociated and eluted as a single low molecular weight (LMWt) peak (Fig. 1c) that had an A260/280 ratio of 0.57, indicating that the protein is essentially nucleic acid free. Multi-angle light scattering (MALS) showed that the rA3G-CD1 fraction from the LMWt peak has a molecular weight of 46.5 kDa, indicating a dimer formation of the 23 kDa monomeric protein (Supplementary Fig. 2a, top).

We further characterized the biochemical properties of the purified rA3G-CD1. rA3G-CD1 showed no detectable deamination activity on ssDNA with CC or TC hotspots, whereas the catalytically active hA3G-CD2/rA3G-CD2 and hA3F-CD2 domains showed deamination products of CC and TC hotspots, respectively (Fig. 1d). Nucleic acid binding by gel-shift assays showed that rA3G-CD1 generated discretely shifted bands of the protein–ssDNA or protein–RNA complexes (Fig. 1e), whereas no shifted band of the protein–nucleic acid complex was detected for hA3G-CD2 at the comparable protein concentration range (Supplementary Fig. 3). These results indicate that rA3G-CD1 is catalytically inactive and has much higher binding affinity to ssDNA/RNA than hA3G-CD2.

### Crystal structure of rA3G-CD1

We obtained two crystal forms of the rA3G-CD1 protein under different crystallization conditions, and determined the structures to 2 Å (CD1-1) in one crystal form and 2.9 Å (CD1-2) in the other crystal form, each in different space groups and with different crystal packing interactions (Table 1). However, these two structures are essentially the same, with a Cα r.m.s. deviation of about 0.54 Å (Supplementary Fig. 2c), indicating that the structures from the two different crystal forms, including the surface loops, are not affected by different crystal packing or buffer conditions. Therefore, the 2 Å high-resolution structure CD1-1 was used for structural analysis and comparison with other A3s, as similar results were also seen with the 2.9 Å resolution structure CD1-2.

**Figure 2:**
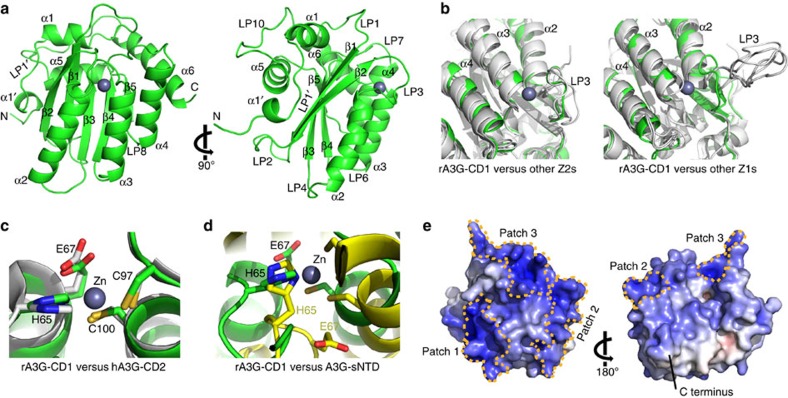
The crystal structure of rA3G-CD1. (**a**) Two views of the 2 Å monomer structure of rA3G-CD1, related by 90° rotation. Secondary structure nomenclature is as described previously^64^. (**b**) Structural superimposition of rA3G-CD1 (green) with other A3s (grey), with focus on the comparison with the longer loop-3 of Z1 group (right) and the shorter loop-3 of Z2 group (left). Other Z1s refer to A3G-CD2 and A3A (3IQS^15^/3IR2 (ref. 34), 4XXO (ref. 39)); and other Z2s refer to A3C (3VOW^35^) and A3Fc-CD2 (4J4J^36^). The A3B-CD2 structure with loop-3 deletion^41^ is not included. (**c**) The Zn-centre superimposition of rA3G-CD1 (green) with catalytically active hA3G-CD2 (3IQS^15^, grey). (**d**) The Zn-centre superimposition of rA3G-CD1 (green) with the A3G-sNTD NMR structure (2MZZ^32^, yellow). (**e**) Surface electrostatic potential of a monomeric rA3G-CD1. The accessible surface area of rA3G-CD1 coloured according to calculated electrostatic potential from −10 *kT*/*e* (red) to 10 *kT*/*e* (blue). Three major positively charged surface patches (patches 1–3) are encircled with yellow dashed lines.

The crystal structure of rA3G-CD1 has the same structural fold as other known A3 domain structures, which are all composed of six major α helices and five core β strands (Fig. 2a). Superimpositions of the crystal structure of rA3G-CD1 with those of A3C^35^, A3F-CD2 (refs 36, 37, 43), A3G-CD2 (refs 15, 34), A3B-CD2 (ref. 41) and A3A^39^ show an overall Cα r.m.s. deviations of 1.23 Å, 1.47/1.73/1.66 Å, 1.46/1.57 Å, 1.34 Å and 1.54 Å, respectively, indicating that rA3G-CD1 shares the conserved core structure of A3 family (Supplementary Fig. 4a). The loop-3 of the Z1 group (A3G-CD2 and A3A) is much longer than that of the Z2 group (rA3G-CD1, A3C and A3F-CD2; Fig. 2b). In fact, the structure around the Zn-centre of rA3G-CD1, which has well-defined electron density for all the residues (Supplementary Fig. 4b), can be superimposed very well with those from the catalytically active hA3G-CD2 and all other available crystal structures of A3s (Fig. 2c; Supplementary Fig. 4c), indicating that there is a strong structural conservation between the catalytically inactive CD1 and other active A3 domains (CD2s and single-domain A3A/A3C). For comparison, the superimposition of the rA3G-CD1 with one of the low energy models of the A3G-sNTD NMR structure^32^ (overall Cα r.m.s. deviation of 2.89 Å) reveals marked differences of the Zn-centre conformation between A3G-sNTD and rA3G-CD1 (and hence other known A3 structures) (Fig. 2d), which probably resulted from the introduced loop-3 and α2 mutations in A3G-sNTD (Supplementary Fig. 4d,e). This large deviation of the Zn-centre conformation between the A3G-sNTD NMR structure and other known A3 structures was also noted in the recent report^32^.

Despite the close similarity of its core structure to other known A3 structures, rA3G-CD1 has a much more pronounced positively charged surface than any other A3 (Fig. 2e; Supplementary Fig. 5). This is consistent with the theoretical isoelectric point (pI) of rA3G-CD1, which is calculated to be relatively high (pI 9.6), similar to hA3G-CD1 (pI 9.4) but very different from hA3G-CD2 (pI 5.81), A3G-sNTD (pI 7.0) and other previously solved A3s (pI 5.0–7.6). Interestingly, the positively charged residues of rA3G-CD1 are mainly distributed on one side of the molecule that is opposite the C terminus (Fig. 2e). Three major positively charged patches are formed around β2-loop-3-α2-α3 (Patch 1), loop-1-loop-7-α6 (Patch 2) and N-α1-loop-10 (Patch 3), respectively (Fig. 2e; Supplementary Table 1). A structure model of the closely related hA3G-CD1 based on rA3G-CD1 also shows similar surface charge features (Supplementary Fig. 5b). In contrast, the NMR structure of A3G-sNTD shows distinctly different surface charge and structural features when compared to rA3G-CD1 (Supplementary Fig. 5c).

### Co-crystal structure of rA3G-CD1 in complex with ssDNA

As the CD1 domain is the major nucleic acid binding domain and has been shown to enhance the overall ssDNA binding and deaminase activity of full-length A3G, we screened for crystal formation of the rA3G-CD1 in complex with ssDNA oligonucleotides of various sequences. A co-crystal form was subsequently obtained in the presence of a 10 nt poly-dT substrate (CD1–dT_10_). The co-crystal of this CD1–dT_10_ complex has the same space group with similar cell dimensions as the apo crystal form of CD1-1 (Table 1).

**Figure 3:**
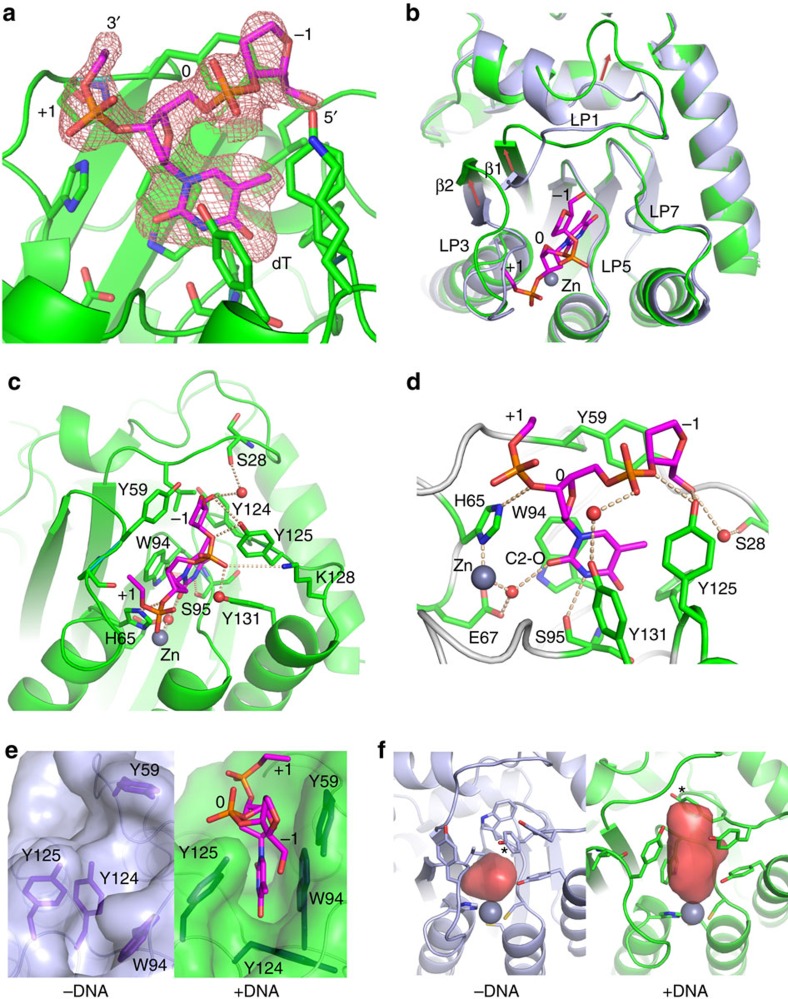
The co-crystal structure of rA3G-CD1 in complex with a poly-dT ssDNA. (**a**) The electron density map (2FoFc, contoured at 1 σ level) for the three nucleotides of the bound poly-dT, which corresponds to one complete dT nucleotide at position 0, the phosphor-backbone/sugar residue at the −1 (5′-end), and the phosphor-backbone at the +1 (3′-end) positions. The amino acid residues important for binding poly-dT are shown in sticks. (**b**) Superimposition of the apo (light blue) and ssDNA co-crystal (green) structures of rA3G-CD1. The nucleotides of the bound ssDNA at the Zn-centre are shown in sticks. Red arrows indicate the major conformational shifts of loop-1/β1 and β2/loop-3 upon DNA binding. (**c**) Detailed bonding interactions between poly-dT and amino acid residues (stick) in the Zn-centre pocket. Zn atom is presented as grey sphere, water molecules as red spheres, hydrogen bonds and electrostatic interactions as dashed lines. (**d**) A zoom-in view of poly-dT bound to the Zn-centre pocket from **c**. The carbonyl group at C2 (C2–O) of the inserted dT at position 0 is labelled. (**e**) The surface representation of the DNA binding pocket in the apo protein (left) and upon ssDNA binding (right). Y59 (loop-3), W94 (loop-5) and Y124 (loop-7) shown in sticks change conformations markedly to accommodate the inserted dT residue. (**f**) Comparison of the Zn-centre pocket volume of rA3G-CD1 before and after binding to poly-dT ssDNA. Red surfaces represent the DNA binding pockets. Star indicates residue Y124.

In the co-crystal structure, we observed clear extra electron density within the pocket around the Zn-centre (Zn-centre pocket). The electron density corresponds to three residues of the bound poly-dT DNA, with defined density for one complete thymine residue (dT) and partially defined density for two flanking residues on its 5′ and 3′ ends (Fig. 3a; Supplementary Fig. 6a). The rest of the ssDNA has no well-defined electron density, likely because any binding to the protein outside the Zn-centre pocket is not very specific or stable.

Compared with the apo structure, the loop-1, loop-3, loop-5, loop-7, β1 and β2 regions around the Zn-centre pocket in the co-crystal structure all have significantly conformational changes (Fig. 3b–f; Supplementary Fig. 6b,c), whereas the rest of rA3G-CD1 is essentially the same. The ssDNA binding around the Zn-centre generates a wide ∼300 Å^3^ pocket that is only about 100 Å^3^ in the apo structure (Fig. 3f). Here, β1 and β2 have shifted away from the Zn-centre by ∼25°, allowing loop-1 and loop-3 to adopt more open conformations (Fig. 3b; Supplementary Fig. 6c).

Interestingly, the loop-7 residue Y124 deep within the Zn-centre pocket has a major role in the binding of rA3G-CD1 to the inserted nucleotide: it seemingly works as a ‘molecular switch' to regulate the ‘open' (with ssDNA) and ‘closed' (without ssDNA) status of the Zn-centre pocket (Fig. 3e,f). Indeed, a Y124A point mutation disrupted binding to both 10 nt poly-dT ssDNA (Supplementary Table 2; Supplementary Fig. 7a,c) and RNA (Supplementary Table 2; Supplementary Fig. 7d,f). The loop-5 residue W94 near the bottom of the Zn-centre pocket, which is in the AID/APOBEC signature motif SWSPC (Supplementary Fig. 1, indicated by stars), flips ∼180° to stack directly with the inserted dT base (Fig. 3c–e). The loop-3 residue Y59 forms a stacking interaction with the deoxyribose backbone at the −1 position on the 5′ end (Fig. 3c–e). The residues S28 (on loop-1), H65 (the Zn-coordinating histidine), Y125 (on loop-7) and Y131 (at the beginning of α4) all form hydrogen bonds with the oligonucleotide phosphate backbone either directly or through mediation by water (Fig. 3c,d). The K128 (on loop-7) also has electrostatic interactions with the phosphate backbone of the ssDNA (Fig. 3c). For the thymine base inserted into the Zn-centre pocket, the oxygen at C2 forms a hydrogen bond with the water molecule that coordinates with the Zn atom, and the nitrogen atom at C3 forms a hydrogen bond with S95 on loop-5 (Fig. 3c,d). Finally, the residues F21, R24 and W34 around loop-1 and residue R122 at the beginning of loop-7, together with their associated loops, further show marked conformational changes upon binding to ssDNA (Supplementary Fig. 6b). Collectively, the structure of rA3G-CD1 in complex with poly-dT reveals the conformational changes of the ssDNA-binding pocket around the Zn-centre and the detailed bonding interactions between the residues on loops 1, 3, 5 and 7 with ssDNA.

### Identification of dimer interface of rA3G-CD1 in solution

**Figure 4:**
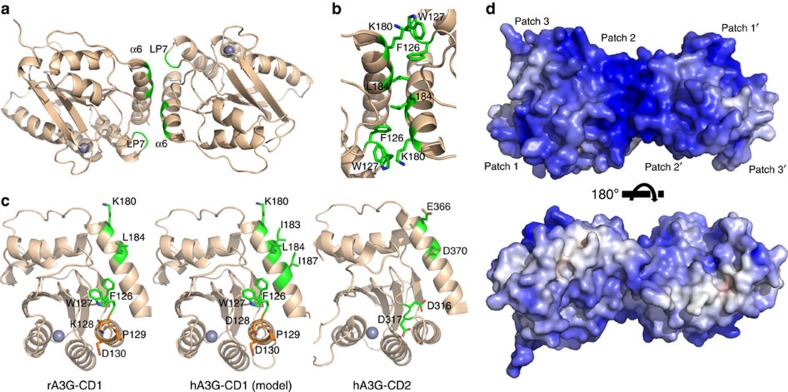
The dimerization of rA3G-CD1. (**a**) The rA3G-CD1 dimer is formed through the interactions mediated by α6 and loop-7 (LP7). (**b**) Key residues critically involved in dimerization interface are shown in green sticks. (**c**) The locations of the key residues (green) for the CD1–CD1 dimerization on the surface of rA3G-CD1 (left) and the hA3G-CD1 (middle, modelled by rA3G-CD1 structure). The same orientation for hA3G-CD2 (3IQS^15^, right) is shown for comparison. The 128–130 KPD/DPD residues involved in Vif and A3G-CD1 interactions are coloured in orange. (**d**) Two views of the surface electrostatic potential of a rA3G-CD1 dimer, showing extensive positive charge (blue) on one side of the dimer. The top panel is the same orientation as in panel-a. The accessible surface is coloured by calculated electrostatic potential from −10 *kT*/*e* (red) to 10 *kT*/*e* (blue).

Since purified rA3G-CD1 exists primarily as a dimer in solution, we attempted to identify the dimerization interface. Examination of the monomer–monomer interfaces present in the two crystal forms revealed 10 molecular interfaces. Interestingly, only one interface is shared between the two different crystal forms (Fig. 4a), which has the largest contact surface area of ∼726 Å^2^ (compared to other non-shared interfaces ranging from 664 to 88 Å^2^), suggesting that this shared interface is the dimer interface. The interactions of this shared interface are mediated through residues coming from loop-7 and α6 of the two subunits, arranged in a tail–tail (or C–C terminus) configuration (Fig. 4a). Several residues centred around L184 on α6 from each subunit pack with each other, while W127 on loop-7 of one CD1 subunit packs with the aliphatic side-chain of K180 on α6 of another CD1 subunit (Fig. 4b). The loop-7 residue F126 is buried within the interfaces, forming the hydrophobic stacking interactions between loop-7 and α6 (Fig. 4b).

To verify the dimerization interactions at this shared interface, we made a quadruple mutant containing F126Y, W127S, K180S, L184S (FWKL) within this interface. This FWKL mutant effectively converts the WT dimer (46.5 kDa) into a clean 27.1 kDa species that is close to the theoretical 23 kDa of a monomer (Supplementary Fig. 2a, bottom), demonstrating that this tail–tail interface is responsible for the dimer formation of rA3G-CD1. These FWKL residues are also conserved in hA3G-CD1, but not in either CD2 domain (Fig. 4c, yellow-highlighted residues in Supplementary Fig. 1), suggesting the conservation of dimerization interface between rA3G-CD1 and hA3G-CD1. In addition, hA3G-CD1 has two more hydrophobic residues (I183 and I187) near the L184 on the α6–α6 interface, making this interface even more hydrophobic in hA3G than rA3G (Fig. 4c). A multiple sequence alignment reveals that these dimer interface residues are relatively conserved among the CD1 domains of A3B, A3F and A3D (yellow-highlighted residues in Supplementary Fig. 1), suggesting potentially similar, but non-identical dimerization interfaces across the CD1 domains of other double-domain A3s.

### Dimer formation is important for nucleic acid binding

Interestingly, the purified monomer FWKL mutant of rA3G-CD1 no longer formed HMWt aggregates and was purified as an LMWt form that was free of bound nucleic acids even without PEI treatment, suggesting that the dimerization of rA3G-CD1 is important for nucleic acid binding and the associated oligomerization. The formation of the rA3G-CD1 dimer merges the positively charged patch 2 (Fig. 2e) of the two CD1s and bridges the positively charged patch 1 and patch 3 on the same side of the dimer, generating a wide positively charged surface (Fig. 4d). This expanded positively charged surface suggests that the dimer may have stronger interactions with the negatively charged nucleic acids than the monomer form. Isothermal titration calorimetry (ITC) binding assays showed that the rA3G-CD1 WT dimer bound to the 10 nt poly-dT ssDNA and 10 nt RNA, whereas the FWKL mutant had no detectable binding to the same substrates (Supplementary Table 2; Supplementary Fig. 7a,b,d,e). ITC assays with longer ssDNA and RNA substrates also showed clear differences in binding patterns and enthalpy changes between WT and the FWKL mutant. The binding data for these long oligonucleotides cannot fit into simple binding models, and may suggest multiple binding sites both on the longer DNA and RNA strands as well as on the proteins (Supplementary Fig. 7g–l). Consistent with the results of ITC, gel-shift assays showed that, unlike the discrete shifted bands of the ssDNA/RNA–protein complexes for WT (Fig. 1e), the FWKL mutant had only gradual changes on the band migration at high protein concentration range (Supplementary Fig. 3), further suggesting weak interactions of the mutant protein with nucleic acids. Interestingly, ITC showed that the stoichiometry of 10 nt RNA (*n*=0.83) is smaller than 10 nt poly-dT ssDNA (*n*=1.47), suggesting that the rA3G-CD1 may have a stronger binding to RNA than the ssDNA of the same length; this is consistent with what was observed in the gel-shift assays with 50 nt DNA/RNA (Fig. 1e).

### Dimerization interactions critical to hA3G-Vif interaction

**Figure 5:**
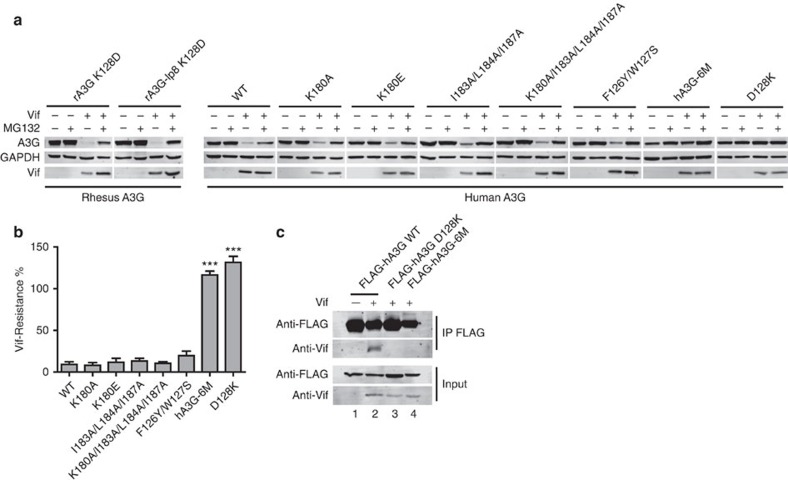
Important hA3G residues for HIV-1 Vif-mediated degradation. (**a**) Vif-mediated degradation of A3G. MG132 is indicated. Left panel, degradation assay of rA3G K128D, and rA3G-lp8 K128D (containing the loop-8 mutations as in the structure of rA3G-CD1). Right panel, degradation assay of hA3G mutants with different combination of point mutations of the six key residues (F126Y, W127S, K180A, I183A, L184A, I187A) at the dimerization interface, WT and D128K mutants are positive and negative controls, respectively. hA3G-6M refers to F126Y/W127S/K180A/I183A/L184A/I187A. In each case, a representative western blot image (of three independent cell-based assays) was chosen for the figure. (**b**) Quantification of the Vif-mediated degradation assay results of dimerization interface mutants from **a**. The percentage of Vif-resistance was calculated by reduction for the hA3G protein level in the presence versus in the absence of Vif, without addition of MG132. The averaged values (of three independent cell-based assays) with s.e.m. are shown in the bar graph. *P* values by one-way *t*-test of each mutant versus WT were calculated. ***P<0.001. (**c**) Co-immunoprecipitation of hA3G WT, D128K and hA3G-6M with Vif.

A single K128D mutation on rA3G-CD1 has been shown to be sufficient to allow for degradation by HIV-1 Vif^27^,^28^,^29^,^30^. We obtained the humanized K128D mutants of full-length (FL) WT rA3G and a FL mutant with the same loop-8 mutation as in the rA3G-CD1 structure (rA3G-lp8). Similar to hA3G, both FL rA3G K128D mutants are susceptible to HIV-1 Vif-mediated degradation (Fig. 5a, left panel), suggesting that rA3G and hA3G share the conserved Vif-binding interface except at the position of the residue 128 on CD1, and that the loop-8 mutation of rA3G-CD1 does not affect A3G–Vif interaction. Since the residues involved in dimerization of rA3G-CD1 are close to the critical D128 residue and are also conserved in hA3G-CD1 (Fig. 4c), we tested whether this dimerization interface is important for Vif-hA3G interaction inside cells using co-immunoprecipitation. We co-expressed HIV-1 Vif with FLAG-tagged hA3G WT, hA3G-D128K and a hA3G dimerization-deficient mutant (F126Y/W127S/K180A/I183A/L184A/I187A, refers as hA3G-6M) in HEK293T cells in the presence of MG132, which inhibits proteasome-mediated degradation of A3G but does not inhibit the binding of Vif to A3G. Vif was co-precipitated with hA3G WT, but not with hA3G-D128K (negative control) and hA3G-6M (Fig. 5c), indicating that hA3G-6M dimerization-deficient mutant does not interact with Vif.

We further evaluated the susceptibility of this dimerization-deficient mutant to Vif-mediated degradation. The percentage of hA3G protein reduction level in the presence and the absence of HIV-1 Vif were evaluated and defined as the Vif-resistance level^35^. Consistent with previous reports^25^,^26^, the majority of hA3G WT was degraded but A3G-D128K was completely resistant to degradation (Fig. 5a,b). Similar to A3G-D128K, A3G-6M was also resistant to Vif-mediated degradation (Fig. 5a,b). Interestingly, any partial mutation of these dimerization-related residues only showed a very minor effect on Vif-resistance (Fig. 5a,b). These findings suggest that the residues for the dimerization interactions are critical for the Vif–A3G interaction and Vif-mediated degradation of A3G.

## Discussion

In this study, we report the high-resolution crystal structure of the N-terminal CD1 domain from rA3G. Similar to hA3G-CD1, rA3G-CD1 forms oligomers, binds to ssDNA/RNA strongly, and is catalytically inactive. A single K128D mutation enables rA3G to be targeted by HIV-1 Vif for proteasomal degradation^29^ (Fig. 5a, left panel), confirming that hA3G-CD1 and rA3G-CD1 likely share conserved Vif–A3G interface interactions with the exception of D/K128. Previous studies demonstrated that rA3G K128D mutant is capable of inhibiting HIV-1 infection at a similar level as hA3G in living cells^29^, providing evidence for the conserved antiviral and Vif-interaction features between rA3G and hA3G. Thus, highly conserved biochemical and biological functions between CD1s of human and rhesus A3Gs indicate that the structure of rA3G-CD1 provides adequate ground for structural study of hA3G-CD1.

We also report the co-crystal structure of rA3G-CD1 bound to a poly-dT ssDNA, which provides a molecular view of how APOBEC binds to ssDNA. It has been challenging to crystallize an APOBEC–ssDNA complex, possibly due to the weak interactions between APOBECs and ssDNA substrates; the flexibility of a ssDNA molecule, combined with a preferred (but not mandatory) DNA sequence specificity to degenerative di- or tri-nucleotide motifs for deamination, make stabilizing the protein–substrate complex difficult. In fact, some APOBEC proteins have been proposed to bind ssDNA dynamically so it can act on different regions along the same ssDNA strand^9^,^44^,^45^. We found that rA3G-CD1 has relatively strong binding affinity to ssDNA, potentially due to its extensively positively charged surface. A3G has been reported to bind to poly-dT oligonucleotide more efficiently than other oligonucleotides^10^, which may explain why we were able to obtain the co-crystal with the poly-dT ssDNA after trying different oligonucleotides.

The co-crystal structure shows clear electron density of three nucleotide residues, with the complete density for one dT residue inserted into the Zn-centre, and partial density for the two flanking residues, which form specific bonding interactions with loop-1, 3, 5 and 7. The electron density for the rest of the ssDNA residues is not sufficiently featured to build DNA with confidence, indicating that their interactions with the protein are either not sufficiently strong or not specific. Thus, this observation from the co-crystal structure may reflect the real nature of how rA3G-CD1 (or an APOBEC protein in general) interacts with a ssDNA substrate. The well-defined density for the central dT nucleotide and the backbones of the two flanking residues observed in the Zn-centre pocket indicates that this part of ssDNA is bound tightly with specific bonding interactions, whereas the distal part of the DNA may be mostly making non-specific charge–charge contacts, possibly in a more dynamic manner. This may provide a structural rationalization for the observed processivity of A3G catalysis along ssDNA, which is a property that is mainly mediated through CD1 domain^9^,^14^,^15^.

The 2 Å crystal structure of rA3G-CD1 indicates that the core structures of both the non-catalytic CD1 and the catalytic domains of A3s are highly conserved, including the Zn-centre conformation (Fig. 2c; Supplementary Fig. 4c), the core β-strands (Supplementary Fig. 4a), the N-terminal end of α2 and the orientations of α2–α4 (Supplementary Fig. 4a,d,e). For comparison, the recently reported NMR structure of A3G-sNTD shows very different conformation of these structural elements (Fig. 2d; Supplementary Fig. 4a,d,e)^32^. On the basis of this NMR structure, a smaller Zn-centre pocket than A3G-CD2 was proposed to explain the lack of sufficient space for accommodating a nucleotide and thus lack of catalytic activity in A3G-CD1 (ref. 32). However, this small Zn-centre pocket may be due to the short length of loop-3, since the apo structures of the catalytically active A3C and A3F-CD2—both with a similarly short loop-3 length—also show a relatively small Zn-centre pocket^46^. The rA3G-CD1 structures here reveal that the volume of the small Zn-centre pocket in the apo structure expands by threefold in the ssDNA complex structure to accommodate the dT residue, which is accomplished through major conformational changes involving loop-1, 3, 5, 7 and the ends of β1 and β2 upon ssDNA binding.

Considering the sizes of thymine and cytosine are similar, the structure of rA3G-CD1 bound to poly-dT may explain the lack of catalytic activity of CD1. Previous studies on the free nucleotide cytidine deaminase suggest that the catalytic activity of a deaminase requires the N3 and C4 amine of the cytidine to envisage to the active centre E67 residue and Zn^47^. However, in the co-crystal structure of rA3G-CD1 with poly-dT, the C2 carbonyl group instead points to the E67 and Zn (Fig. 3d), and the N3 and C4 of the base are too far away from the E67/Zn for deamination to occur. The residues on loop-1 and loop-3 of rA3G-CD1 are different from those of the active A3 domains, which could be responsible for generating the base orientation that is not suitable for deamination. A co-crystal structure of a catalytically active APOBEC domain with ssDNA will help resolve this intriguing question.

Previous studies proposed several ssDNA-binding models around the Zn-centre of the catalytically active APOBECs^15^,^33^,^36^,^38^,^48^,^49^,^50^,^51^. Considering the highly conserved key residues around the Zn-centre pocket between catalytic domains and non-catalytic CD1 domains, it is likely that the interactions between these conserved residues and the ssDNA poly-dT observed in rA3G-CD1 might be similar in other AID/APOBEC proteins. The SWSPC is the signature motif that is exclusively conserved in most of AID/APOBEC but not in other deaminases^52^ (Supplementary Fig. 1). Here, we show that the tryptophan in this motif (in this case, W94 in rA3G-CD1) has a critical role in stacking with the inserted base. The loop-7 residue Y124 acts as a ‘molecular switch' that open/close the Zn-centre pocket upon ssDNA binding. These characteristics are consistent with that the mutations of W94 and Y124 equivalents in other A3s significantly eliminate nucleic acid binding ability and catalytic activity^15^,^33^,^36^,^41^,^48^. Recently the crystal structure of A3B-CD2 bound to a free nucleotide dCMP was reported^41^. In this structure the dCMP is not bound to the Zn-centre pocket (Supplementary Fig. 6d) and the location of the bound free dCMP is not near the 5′ or 3′ end of the three nucleotides from the rA3G–CD1–ssDNA co-crystal structure (Supplementary Fig. 6e).

One of the biologically relevant features of A3G is oligomerization. hA3G is isolated as megadalton high-molecular-mass (HMM) complexes^9^,^53^. After RNase A treatment, the HMM can be converted into smaller low-molecular-mass (LMM) complexes^9^,^53^. Similar to hA3G, rA3G-CD1 forms HMWt aggregates that can be converted into an LMWt species after removal of nucleic acids by PEI. The LMWt rA3G-CD1 is dimeric, suggesting that the CD1–CD1 dimeric form is capable of aggregating into HMWt complexes through binding to nucleic acids. Although the possibility of CD2–CD2 dimerization of A3G has also been proposed by previous studies^54^, considering that purified A3G-CD2 alone exists only as a monomeric form^33^ and does not show strong binding to nucleic acids, the oligomerization of A3G is likely primarily mediated by its CD1 that dimerizes and binds to nucleic acids.

The strong nucleic acid binding ability of rA3G-CD1 could potentially be enhanced by dimer formation through the generation of contiguous positively charged patches. This implies that a longer ssDNA/RNA may bind to both subunits of a CD1 dimer through charge–charge interactions across the positively charged patch 2 surfaces near α6 and loop-7. The R24, W94, Y124 and W127 residues of hA3G-CD1 have been reported to be important for RNA binding and A3G oligomerization^13^,^55^,^56^. These four residues from either subunit of the dimer are positioned right near each other along the positively charged patch 2 (Supplementary Figs 8a,9). A recent study of crosslinking hA3G with DNA/RNA shows that α6 of CD1 is also involved in interacting with nucleic acids^57^. The structure of rA3G-CD1 bound to a 10 nt poly-dT reported here suggests that the site-specific interaction within the Zn-centre pocket may also contribute to the overall ssDNA binding. Interestingly, in this co-crystal structure, we observed that in the rA3G-CD1 dimer only one Zn-centre pocket binds to the DNA, whereas the other Zn-centre pocket is DNA free and keeps the same conformation as the apo structure. Although this may be due to spatial constraint by the crystal packing, considering the directionality of the bound ssDNA within the Zn-centre pocket, it is also possible that one ssDNA molecule only binds to one Zn-centre pocket in a rA3G-CD1 dimer.

Vif-mediated degradation is a mechanism for HIV-1 to counteract A3G, and so the A3G–Vif interface is an important therapeutic target for protecting A3G and its anti-HIV activity. Previous studies reported that hA3G mutations on DPD 128–130 are resistant to Vif-mediated degradation^25^,^26^,^31^. Switching the charge at D/K128 between human and rhesus A3G determines the species-specific Vif susceptibility, suggesting that the local electrostatic interaction has the key role in A3G–Vif interaction. Recently a study based on patient-derived Vif variants and HIV-forced evolution identified a new hA3G mutant Y125R that is resistant to Vif-mediated degradation and mapped the interactions between Y125, D128, D130 of hA3G and HIV-1 Vif^31^. In the crystal structure of rA3G-CD1, Y125, K128 and D130 are in close proximity, forming a compact charged interface around residue 128 (Supplementary Fig. 8b). The hA3G-CD1 structure model shows similar structural characteristics but with a completely reversed local charge due to the Lys to Asp conversion at residue 128 (Supplementary Fig. 8c). This structural feature offers a plausible explanation for the previous understanding of the key role D/K128 has in Vif binding by hA3G and rA3G, respectively. The residue F126 is also reported as being important for Vif-mediated degradation as A3G-sNTD is resistant to Vif and mutating F126A back to phenylalanine restores Vif susceptibility^32^. However, F126A has not been reported to be Vif-resistant in the study of WT A3G^25^. Although the mutated residue F126A in the NMR structure of A3G-sNTD is solvent-exposed, in all other known A3 structures with a WT loop-7, including rA3G-CD1 structures with or without bound to poly-dT, this conserved phenylalanine is relatively found towards the interior of the protein. Thus, F126 of WT A3G is not likely to directly interact with Vif.

In addition to the residues discussed above, several residues located on loop-1, loop-3 and loop-7 of A3G-sNTD were reported to have a role in interacting with HIV-1 Vif^32^. A mutagenesis study on WT hA3G identified a similar interface consisting of loop-1, loop-5 and loop-7 residues that are involved with both A3G–Vif binding and A3G–A3G interactions^26^, suggesting that there may be an association between A3G oligomerization and interaction with Vif. On the basis of the crystal structure of the rA3G-CD1 dimer and further mutational studies, we showed that the CD1–CD1 dimerization may indeed have a critical role in A3G–Vif interaction and Vif-mediated degradation, as hA3G becomes almost 100% resistant to HIV-1 Vif after mutating residues to disrupt the dimerization interface. Interestingly, hA3G carrying partial mutations at this dimer interface is still subjected to degradation, probably because these mutants are not monomeric in a cellular environment and thus are still sensitive to Vif-mediated degradation. Our findings raise a new question regarding to the relationship between A3G dimerization and HIV-1 Vif targeting, which is intriguing for future studies.

In summary, we report the crystal structures of A3G-CD1 from rhesus macaque alone and in complex with a poly-dT ssDNA. Structural comparison between the apo and DNA-bound forms of rA3G-CD1 unravels the ssDNA binding-related conformational changes and the details of interactions between ssDNA and the Zn-centre pocket, which may be highly conserved within the APOBEC/AID family. We have identified a dimerization interface that is important for nucleic acid binding and oligomerization of rA3G-CD1, and demonstrated that this dimerization interface is critical to Vif-binding and Vif-mediated degradation of hA3G. These results would facilitate the further functional study of A3G and the development of anti-HIV strategies by blocking Vif-mediated degradation of A3G.

## Methods

### Protein expression and purification

All APOBEC protein encoding sequences were cloned into a pGEX-6P-1 vector (GE Healthcare) with an N-terminal GST tag and PreScission cleavage site. *E. coli* cells transformed with the plasmids were grown in LB media at 37 °C until the OD_600_ reached 0.6. Cultures were then reduced to 14 °C and induced with 0.2 μM IPTG overnight. The collected bacteria cell pellets were resuspended in buffer A (500 mM NaCl, 50 mM HEPES pH 7.5, 1 mM DTT) with 0.1 mg ml^−1^ RNase A (Qiagen) and lysed by a microfluidizer system. After centrifugation, the supernatant of the cell lysates were incubated with glutathione resin (GE Healthcare), and washed with four column volumes of buffer A with 500 mM NaCl, and overnight digestion by PreScission protease in buffer B (500 mM NaCl, 50 mM HEPES pH 7.5, 1 mM TCEP). The elution of the cleaved rA3G-CD1 protein was treated with PEI (0.1%) until no more white precipitation formed. After centrifugation, the supernatant was analyzed by Superose 6 10/300 GL (GE Healthcare), and further purified by Fast Flow (GE Healthcare) HiLoad 16/60 Superdex 75 gel filtration in large-scale. The rA3G-CD1 FWKL and Y124A mutant, hA3G-CD2 and rA3G-CD2 were purified directly by HiLoad 16/60 Superdex 75 gel filtration (GE Healthcare) without PEI treatment. A hA3F-CD2 construct retaining the GST tag was purified by the same method without PreScission cleavage and used for the deamination assay. Purified protein samples were analyzed by SDS–PAGE, and stained with Coomassie blue.

### Protein crystallization and data collection

Purified rA3G-CD1 protein was concentrated to 10 mg ml^−1^. Optimized crystals were obtained by a hanging drop vapor-diffusion method in 0.2 M Na K tartrate, 15% PEG 4000 after one week (CD1-1), and in 1.9 M AmSO_4_, 0.1 M Tris pH 8.0, 0.5 M NDSB-195 after 1  month (CD1-2). For co-crystallization with poly-dT, 10 nt poly-dT synthesized from Integrated DNA Technologies (IDT) was mixed with protein in a 1.2:1 ratio for crystallization screening. Protein–DNA co-crystals were obtained at 0.1 M Tris pH 8.5, 8% PEG 8000 and further optimized at 0.1 M Tris pH 8.0, 6% PEG 8000. Diffraction data was collected from Advanced Light Source BL-8.2.1 and Advanced Photon Source 19-ID/23-ID.

### Structure determination and refinement

A complete data set for CD1-1 was collected, indexed, integrated and scaled by the HKL2000 program package. The structure of CD1-1 was determined by molecular replacement (MolRep, CCP4 suite) using A3Fc-CD2 (PDB: 4J4J) as a template. The structure was then refined by PHENIX and rebuilt in COOT. The structure of CD1-2 and the co-crystal structure of CD1–dT_10_ were determined by the same method using CD1-1 as the molecular replacement template. The ssDNA was built manually and verified by omit map. The statistics for diffraction data and structural determination/refinement for all structures are shown in Table 1.

### Structural modelling, comparison and analysis

The structure of hA3G-CD1 was modelled by Phyre2 (ref. 58) with a one-to-one threading method, using the 2 Å structure of rA3G-CD1 (CD1-1) as a template. Surface electrostatic potential of rA3G-CD1 and other A3s was calculated by APBS^59^. Structure comparison and crystallographic interfaces were analyzed by PDBeFold^60^ and PDBePISA^61^. Pockets were analyzed and visualized by HOLLOW^62^.

### Multi-angle light scattering (MALS)

Experiments were conducted at the University of Southern California NanoBiophysics Core Facility. Purified rA3G-CD1 protein and mutants were subjected to HPLC chromatography (Shodex 802.5) in buffer C (250 mM Na_2_SO_4_, 50 mM HEPES pH 7.5, 0.5 mM TCEP). The column effluent was passed directly on-line into Dawn Heleos MALS detector (Wyatt Technology) and Optilab rEX refractometer (Wyatt Technology). Data was analyzed by ASTRA 6 software.

### Deamination and electrophoresis mobility shift assay (EMSA)

For deamination assays, purified rA3G-CD1, hA3G-CD2 and hA3F-CD2 were incubated with 5′-FAM-labelled 30 nt ssDNA (Supplementary Table 2) and 2 U of uracil–DNA glycosylase in the condition of 60 mM HEPES pH 7.5, 50 mM NaCl (10 μl reaction volume), 37 °C for 3 h. The reaction mixture was then mixed with 10 μl of formamide, 25 mM EDTA and 50 mM NaOH. The reaction products were heat at 97 °C for 10 min, and analyzed by native PAGE.

For EMSA, purified rA3G-CD1, rA3G-CD1 FWKL mutant and hA3G-CD2 inactive mutant (E259Q) were incubated with 5′-FAM-labelled 50 nt ssDNA, 50 nt RNA and 10 nt ssDNA poly-dT (Supplementary Table 2) on ice in the condition of 60 mM HEPES pH 7.5, 100 mM NaCl (10 μl reaction volume) for 10 min. The reaction mixture was then mixed with 2 μl of 80% glycerol and analyzed by native PAGE.

### ITC assay

ITC experiments were carried out at 25 °C using a MicroCal PEAQ-ITC system (GE Lifescience). Protein samples of rA3G-CD1, FWKL and Y124A mutants in buffer (100 mM NaCl, 50 mM HEPES pH 7.5) were filled in the sample cell (280 μl volume) and titrated with ssDNA/RNA substrates (40 μl, synthesized and purified by Integrated DNA Technologies, Supplementary Table 2), which were dialyzed (for ssDNA) or dissolved (for RNA) in the same buffer. Protein concentrations were 10 or 25.6 μM for rA3G-CD1, 9.88 μM for FWKL mutant and 4 μM for Y124A, respectively. ssDNA/RNA were at 50–200 μM depending on the saturation points. Enthalpy data was normalized by concentration and processed in MicroCal PEAQ-ITC Analysis software. One set of sites model was used to fit with 10 nt ssDNA/RNA data.

### Vif-dependent degradation assay of APOBEC3G

Human A3G WT and mutants were cloned into a pcDNA 3.1(+) vector (Invitrogen) with an N-terminal FLAG tag. pcDNA-HVif was obtained from the NIH AIDS Reagent Program^63^. pcDNA-FLAG-A3G WT or mutants were co-transfected with pcDNA-HVif or pcDNA 3.1(+) (negative control) by using X-tremeGENE 9 DNA Transfection Reagent (Roche), into HEK293T cells (ATCC) in 12-well plates. Twenty-four hours after transfection, 16 μM of MG132 (Sigma) or DMSO was added and incubated for another 24 h at 37 °C. Cells were then lysed in 1 × RIPA buffer with 1 × complete protease inhibitors (Roche) and subjected to western blot with anti-FLAG M2 mAb (F3165, Sigma, 1:3,000), anti-GAPDH mAb (GTX627408, GeneTex, 1:5,000) and anti-Vif mAb (#319, NIH AIDS Reagent Program, 1:2,000). Quantification of Vif-resistance levels was analyzed based on three independent transfection experiments. The original uncropped scans are in Supplementary Fig. 10.

### Co-immunoprecipitation assay

Transfections of the FLAG-tagged human A3G WT and A3G-6M mutant were performed with the same protocol as in the degradation assays. Twenty-four hours after transfection, cells were treated with 16 μM MG132 for another 24 h. Cells were then lysed in lysis buffer (20 mM HEPES pH 8.0, 150 mM NaCl, 0.5 mM DTT, 1.5 mM MgCl_2_, 1% NP-40) with Benzonase nuclease (Sigma) and 1 × complete protease inhibitors (Roche). Cell lysate was incubated with anti-FLAG M2 agarose (Sigma) at 4 °C overnight. Beads were then washed four times with 1 ml wash buffer (20 mM HEPES pH 8.0, 150 mM NaCl, 0.5 mM DTT, 0.05% NP-40) before eluting with FLAG elution buffer (wash buffer supplemented with 250 μg ml^−1^ of 3 × FLAG peptide (Sigma)). The eluted proteins were analyzed by western blot with the same antibodies as in Vif-dependent degradation assay. The original uncropped scan is in Supplementary Fig. 10.

### Data availability

Atomic coordinates and structure factors for rA3G-CD1 (CD1-1, CD1-2) and rA3G-CD1–ssDNA complex (CD1–dT_10_) have been deposited in the Protein Data Bank (PDB) with the accession codes 5K81, 5K82 and 5K83, respectively. All other data are available from the authors on reasonable request.

## Additional information

**How to cite this article**: Xiao, X. *et al*. Crystal structures of APOBEC3G N-domain alone and its complex with DNA. *Nat. Commun.* 7:12193 doi: 10.1038/ncomms12193 (2016).

## Supplementary Material

Supplementary Information Supplementary Figures 1-10 and Supplementary Tables 1-3

## Figures and Tables

**Table 1:** Data collection and refinement statistics.

	CD1–1	CD1–2	CD1–dT_10_
*Data collection*			
Space group	P1 21 1	P32	P1 21 1
Cell dimensions			
*a*, *b*, *c* (Å)	106.2, 81.3, 106.3	152.4, 152.4 79.8	105.7, 83.3, 105.8
*α*, *β*, *γ* (°)	90, 120, 90	90, 90, 120	90, 120, 90
Resolution (Å)	50–2 (2.07–2.00)	50–2.90 (2.95–2.90)	50–2.39 (2.48–2.39)
*R*_sym_ or *R*_merge_	7.4 (58.3)	11.6 (67.7)	7.6 (43)
*I*/σ*I*	18.6 (2.5)	29.4 (1.9)	26 (4.2)
Completeness (%)	97.8 (82.1)	98.8 (85.7)	100 (99.7)
Redundancy	4.4 (3)	7 (5.3)	6.9 (5)
			
*Refinement*			
Resolution (Å)	46.03–2.00	42.30–2.91	45.79–2.39
No. reflections	102,317	44,680	63,261
*R*_work_/*R*_free_	18.9/22	22.3/25	18.7/25.1
No. atoms	10,141	6,342	9,837
Protein	9,493	6,338	9,454
Ligand/ion	6	4	107
Water	642	0	276
*B*-factors	37.50	90.60	45.40
Protein	37.10	90.60	45.50
Ligand/ion	31.50	84.40	39.90
Water	42.10	NA	41.70
r.m.s. deviations			
Bond lengths (Å)	0.011	0.022	0.009
Bond angles (°)	1.34	1.71	1.17

NA, not applicable; r.m.s., root mean square.

Each structure was determined from a single crystal.

Highest-resolution shell is shown in parentheses.

## References

[b1] LaRue R. S. . Guidelines for naming nonprimate APOBEC3 genes and proteins. J. Virol. 83, 494–497 (2009). 18987154 10.1128/JVI.01976-08 PMC2612408

[b2] Feng Y., Baig T. T., Love R. P. & Chelico L. Suppression of APOBEC3-mediated restriction of HIV-1 by Vif. Front Microbiol. 5, 450 (2014). 25206352 10.3389/fmicb.2014.00450 PMC4144255

[b3] Bransteitter R., Prochnow C. & Chen X. S. The current structural and functional understanding of APOBEC deaminases. Cell. Mol. Life. Sci. 66, 3137–3147 (2009). 19547914 10.1007/s00018-009-0070-y PMC11115857

[b4] Fu Y. . DNA cytosine and methylcytosine deamination by APOBEC3B: enhancing methylcytosine deamination by engineering APOBEC3B. Biochem. J. 471, 25–35 (2015). 26195824 10.1042/BJ20150382 PMC4613526

[b5] Sheehy A. M., Gaddis N. C., Choi J. D. & Malim M. H. Isolation of a human gene that inhibits HIV-1 infection and is suppressed by the viral Vif protein. Nature 418, 646–650 (2002). 12167863 10.1038/nature00939

[b6] Mangeat B. . Broad antiretroviral defence by human APOBEC3G through lethal editing of nascent reverse transcripts. Nature 424, 99–103 (2003). 12808466 10.1038/nature01709

[b7] Yu Q. . Single-strand specificity of APOBEC3G accounts for minus-strand deamination of the HIV genome. Nat. Struct. Mol. Biol. 11, 435–442 (2004). 15098018 10.1038/nsmb758

[b8] Harris R. S. . DNA deamination mediates innate immunity to retroviral infection. Cell 113, 803–809 (2003). 12809610 10.1016/s0092-8674(03)00423-9

[b9] Chelico L., Pham P., Calabrese P. & Goodman M. F. APOBEC3G DNA deaminase acts processively 3′ --> 5′ on single-stranded DNA. Nat. Struct. Mol. Biol. 13, 392–399 (2006). 16622407 10.1038/nsmb1086

[b10] Iwatani Y., Takeuchi H., Strebel K. & Levin J. G. Biochemical activities of highly purified, catalytically active human APOBEC3G: correlation with antiviral effect. J. Virol. 80, 5992–6002 (2006). 16731938 10.1128/JVI.02680-05 PMC1472592

[b11] Navarro F. . Complementary function of the two catalytic domains of APOBEC3G. Virology 333, 374–386 (2005). 15721369 10.1016/j.virol.2005.01.011

[b12] Svarovskaia E. S. . Human apolipoprotein B mRNA-editing enzyme-catalytic polypeptide-like 3G (APOBEC3G) is incorporated into HIV-1 virions through interactions with viral and nonviral RNAs. J. Biol. Chem. 279, 35822–35828 (2004). 15210704 10.1074/jbc.M405761200

[b13] Wang T. . 7SL RNA mediates virion packaging of the antiviral cytidine deaminase APOBEC3G. J. Virol. 81, 13112–13124 (2007). 17881443 10.1128/JVI.00892-07 PMC2169093

[b14] Chelico L., Prochnow C., Erie D. A., Chen X. S. & Goodman M. F. Structural model for deoxycytidine deamination mechanisms of the HIV-1 inactivation enzyme APOBEC3G. J. Biol. Chem. 285, 16195–16205 (2010). 20212048 10.1074/jbc.M110.107987 PMC2871487

[b15] Holden L. G. . Crystal structure of the anti-viral APOBEC3G catalytic domain and functional implications. Nature 456, 121–124 (2008). 18849968 10.1038/nature07357 PMC2714533

[b16] Iwatani Y. . Deaminase-independent inhibition of HIV-1 reverse transcription by APOBEC3G. Nucleic Acids Res. 35, 7096–7108 (2007). 17942420 10.1093/nar/gkm750 PMC2175344

[b17] Bishop K. N., Verma M., Kim E. Y., Wolinsky S. M. & Malim M. H. APOBEC3G inhibits elongation of HIV-1 reverse transcripts. PLoS Pathog. 4, e1000231 (2008). 19057663 10.1371/journal.ppat.1000231 PMC2584787

[b18] Adolph M. B., Webb J. & Chelico L. Retroviral restriction factor APOBEC3G delays the initiation of DNA synthesis by HIV-1 reverse transcriptase. PLoS ONE 8, e64196 (2013). 23717565 10.1371/journal.pone.0064196 PMC3662766

[b19] Marin M., Rose K. M., Kozak S. L. & Kabat D. HIV-1 Vif protein binds the editing enzyme APOBEC3G and induces its degradation. Nat. Med. 9, 1398–1403 (2003). 14528301 10.1038/nm946

[b20] Stopak K., de Noronha C., Yonemoto W. & Greene W. C. HIV-1 Vif blocks the antiviral activity of APOBEC3G by impairing both its translation and intracellular stability. Mol. Cell 12, 591–601 (2003). 14527406 10.1016/s1097-2765(03)00353-8

[b21] Yu X. . Induction of APOBEC3G ubiquitination and degradation by an HIV-1 Vif-Cul5-SCF complex. Science 302, 1056–1060 (2003). 14564014 10.1126/science.1089591

[b22] Mehle A. . Vif overcomes the innate antiviral activity of APOBEC3G by promoting its degradation in the ubiquitin–proteasome pathway. J. Biol. Chem. 279, 7792–7798 (2004). 14672928 10.1074/jbc.M313093200

[b23] Jager S. . Vif hijacks CBF-beta to degrade APOBEC3G and promote HIV-1 infection. Nature 481, 371–375 (2012). 22190037 10.1038/nature10693 PMC3310910

[b24] Zhang W., Du J., Evans S. L., Yu Y. & Yu X. F. T-cell differentiation factor CBF-beta regulates HIV-1 Vif-mediated evasion of host restriction. Nature 481, 376–379 (2012). 22190036 10.1038/nature10718

[b25] Huthoff H. & Malim M. H. Identification of amino acid residues in APOBEC3G required for regulation by human immunodeficiency virus type 1 Vif and Virion encapsidation. J. Virol. 81, 3807–3815 (2007). 17267497 10.1128/JVI.02795-06 PMC1866099

[b26] Lavens D. . Definition of the interacting interfaces of Apobec3G and HIV-1 Vif using MAPPIT mutagenesis analysis. Nucleic Acids Res. 38, 1902–1912 (2010). 20015971 10.1093/nar/gkp1154 PMC2847223

[b27] Bogerd H. P., Doehle B. P., Wiegand H. L. & Cullen B. R. A single amino acid difference in the host APOBEC3G protein controls the primate species specificity of HIV type 1 virion infectivity factor. Proc. Natl Acad. Sci. USA 101, 3770–3774 (2004). 14999100 10.1073/pnas.0307713101 PMC374319

[b28] Mangeat B., Turelli P., Liao S. & Trono D. A single amino acid determinant governs the species-specific sensitivity of APOBEC3G to Vif action. J. Biol. Chem. 279, 14481–14483 (2004). 14966139 10.1074/jbc.C400060200

[b29] Schrofelbauer B., Chen D. & Landau N. R. A single amino acid of APOBEC3G controls its species-specific interaction with virion infectivity factor (Vif). Proc. Natl Acad. Sci. USA 101, 3927–3932 (2004). 14978281 10.1073/pnas.0307132101 PMC374346

[b30] Xu H. . A single amino acid substitution in human APOBEC3G antiretroviral enzyme confers resistance to HIV-1 virion infectivity factor-induced depletion. Proc. Natl Acad. Sci. USA 101, 5652–5657 (2004). 15054139 10.1073/pnas.0400830101 PMC397464

[b31] Letko M., Booiman T., Kootstra N., Simon V. & Ooms M. Identification of the HIV-1 Vif and human APOBEC3G protein interface. Cell Rep. 13, 1789–1799 (2015). 26628364 10.1016/j.celrep.2015.10.068 PMC4670588

[b32] Kouno T. . Structure of the Vif-binding domain of the antiviral enzyme APOBEC3G. Nat. Struct. Mol. Biol. 22, 485–491 (2015). 25984970 10.1038/nsmb.3033 PMC4456288

[b33] Chen K. M. . Structure of the DNA deaminase domain of the HIV-1 restriction factor APOBEC3G. Nature 452, 116–119 (2008). 18288108 10.1038/nature06638

[b34] Shandilya S. M. . Crystal structure of the APOBEC3G catalytic domain reveals potential oligomerization interfaces. Structure 18, 28–38 (2010). 20152150 10.1016/j.str.2009.10.016 PMC2913127

[b35] Kitamura S. . The APOBEC3C crystal structure and the interface for HIV-1 Vif binding. Nat. Struct. Mol. Biol. 19, 1005–1010 (2012). 23001005 10.1038/nsmb.2378

[b36] Siu K. K., Sultana A., Azimi F. C. & Lee J. E. Structural determinants of HIV-1 Vif susceptibility and DNA binding in APOBEC3F. Nat. Commun. 4, 2593 (2013). 24185281 10.1038/ncomms3593 PMC4956467

[b37] Bohn M. F. . Crystal structure of the DNA cytosine deaminase APOBEC3F: the catalytically active and HIV-1 Vif-binding domain. Structure 21, 1042–1050 (2013). 23685212 10.1016/j.str.2013.04.010 PMC3805256

[b38] Byeon I. J. . NMR structure of human restriction factor APOBEC3A reveals substrate binding and enzyme specificity. Nat. Commun. 4, 1890 (2013). 23695684 10.1038/ncomms2883 PMC3674325

[b39] Bohn M. F. . The ssDNA mutator APOBEC3A is regulated by cooperative dimerization. Structure 23, 903–911 (2015). 25914058 10.1016/j.str.2015.03.016 PMC4874493

[b40] Harjes E. . An extended structure of the APOBEC3G catalytic domain suggests a unique holoenzyme model. J. Mol. Biol. 389, 819–832 (2009). 19389408 10.1016/j.jmb.2009.04.031 PMC2700007

[b41] Shi K., Carpenter M. A., Kurahashi K., Harris R. S. & Aihara H. Crystal structure of the DNA deaminase APOBEC3B catalytic domain. J. Biol. Chem. 290, 28120–28130 (2015). 26416889 10.1074/jbc.M115.679951 PMC4653671

[b42] Burgess R. R. Use of polyethyleneimine in purification of DNA-binding proteins. Methods Enzymol. 208, 3–10 (1991). 1779840 10.1016/0076-6879(91)08003-z

[b43] Nakashima M. . Structural insights into HIV-1 Vif-APOBEC3F interaction. J. Virol. 90, 1034–1047 (2015). 26537685 10.1128/JVI.02369-15 PMC4702671

[b44] Shlyakhtenko L. S. . Nanoscale structure and dynamics of ABOBEC3G complexes with single-stranded DNA. Biochemistry 51, 6432–6440 (2012). 22809226 10.1021/bi300733d PMC3448016

[b45] Pham P., Bransteitter R., Petruska J. & Goodman M. F. Processive AID-catalysed cytosine deamination on single-stranded DNA simulates somatic hypermutation. Nature 424, 103–107 (2003). 12819663 10.1038/nature01760

[b46] Shandilya S. M., Bohn M. F. & Schiffer C. A. A computational analysis of the structural determinants of APOBEC3's catalytic activity and vulnerability to HIV-1 Vif. Virology 471-473C, 105–116 (2014). 25461536 10.1016/j.virol.2014.09.023 PMC4857191

[b47] Teh A. H. . The 1.48 A resolution crystal structure of the homotetrameric cytidine deaminase from mouse. Biochemistry 45, 7825–7833 (2006). 16784234 10.1021/bi060345f

[b48] Mitra M. . Structural determinants of human APOBEC3A enzymatic and nucleic acid binding properties. Nucleic Acids Res. 42, 1095–1110 (2014). 24163103 10.1093/nar/gkt945 PMC3902935

[b49] Lu X. . Crystal structure of DNA cytidine deaminase ABOBEC3G catalytic deamination domain suggests a binding mode of full-length enzyme to single-stranded DNA. J. Biol. Chem. 290, 4010–4021 (2014). 25542899 10.1074/jbc.M114.624262 PMC4326812

[b50] Logue E. C. . A DNA sequence recognition loop on APOBEC3A controls substrate specificity. PLoS ONE 9, e97062 (2014). 24827831 10.1371/journal.pone.0097062 PMC4020817

[b51] Bulliard Y. . Structure-function analyses point to a polynucleotide-accommodating groove essential for APOBEC3A restriction activities. J. Virol. 85, 1765–1776 (2011). 21123384 10.1128/JVI.01651-10 PMC3028873

[b52] Conticello S. G., Thomas C. J., Petersen-Mahrt S. K. & Neuberger M. S. Evolution of the AID/APOBEC family of polynucleotide (deoxy)cytidine deaminases. Mol. Biol. Evol. 22, 367–377 (2005). 15496550 10.1093/molbev/msi026

[b53] Chiu Y. L. . Cellular APOBEC3G restricts HIV-1 infection in resting CD4+ T cells. Nature 435, 108–114 (2005). 15829920 10.1038/nature03493

[b54] Wedekind J. E. . Nanostructures of APOBEC3G support a hierarchical assembly model of high molecular mass ribonucleoprotein particles from dimeric subunits. J. Biol. Chem. 281, 38122–38126 (2006). 17079235 10.1074/jbc.C600253200 PMC1847398

[b55] Huthoff H., Autore F., Gallois-Montbrun S., Fraternali F. & Malim M. H. RNA-dependent oligomerization of APOBEC3G is required for restriction of HIV-1. PLoS Pathog. 5, e1000330 (2009). 19266078 10.1371/journal.ppat.1000330 PMC2646141

[b56] Bulliard Y. . Functional analysis and structural modeling of human APOBEC3G reveal the role of evolutionarily conserved elements in the inhibition of human immunodeficiency virus type 1 infection and Alu transposition. J. Virol. 83, 12611–12621 (2009). 19776130 10.1128/JVI.01491-09 PMC2786736

[b57] Polevoda B. . RNA binding to APOBEC3G induces the disassembly of functional deaminase complexes by displacing single-stranded DNA substrates. Nucleic Acids Res. 43, 9434–9445 (2015). 26424853 10.1093/nar/gkv970 PMC4627094

[b58] Kelley L. A., Mezulis S., Yates C. M., Wass M. N. & Sternberg M. J. The Phyre2 web portal for protein modeling, prediction and analysis. Nature Protocols 10, 845–858 (2015). 25950237 10.1038/nprot.2015.053 PMC5298202

[b59] Baker N. A., Sept D., Joseph S., Holst M. J. & McCammon J. A. Electrostatics of nanosystems: application to microtubules and the ribosome. Proc. Natl Acad. Sci. USA 98, 10037–10041 (2001). 11517324 10.1073/pnas.181342398 PMC56910

[b60] Krissinel E. & Henrick K. Secondary-structure matching (SSM), a new tool for fast protein structure alignment in three dimensions. Acta Crystallogr. Sect. D 60, 2256–2268 (2004). 15572779 10.1107/S0907444904026460

[b61] Krissinel E. & Henrick K. Inference of macromolecular assemblies from crystalline state. J. Mol. Biol. 372, 774–797 (2007). 17681537 10.1016/j.jmb.2007.05.022

[b62] Ho B. K. & Gruswitz F. HOLLOW: generating accurate representations of channel and interior surfaces in molecular structures. BMC Struct. Biol. 8, 49 (2008). 19014592 10.1186/1472-6807-8-49 PMC2603037

[b63] Nguyen K. L. . Codon optimization of the HIV-1 vpu and vif genes stabilizes their mRNA and allows for highly efficient Rev-independent expression. Virology 319, 163–175 (2004). 15015498 10.1016/j.virol.2003.11.021

[b64] Prochnow C., Bransteitter R., Klein M. G., Goodman M. F. & Chen X. S. The APOBEC-2 crystal structure and functional implications for the deaminase AID. Nature 445, 447–451 (2007). 17187054 10.1038/nature05492

