# Supplementary material for: Crystal structures of APOBEC3G N-domain alone and its complex with DNA
Source: Nat Commun. 2016 Aug 2;7:12193. doi: 10.1038/ncomms12193 (PMC4974639; doi:10.1038/ncomms12193)
Supplement: Supplementary Information — Supplementary Figures 1-10 and Supplementary Tables 1-3 [file ncomms12193-s1.pdf]

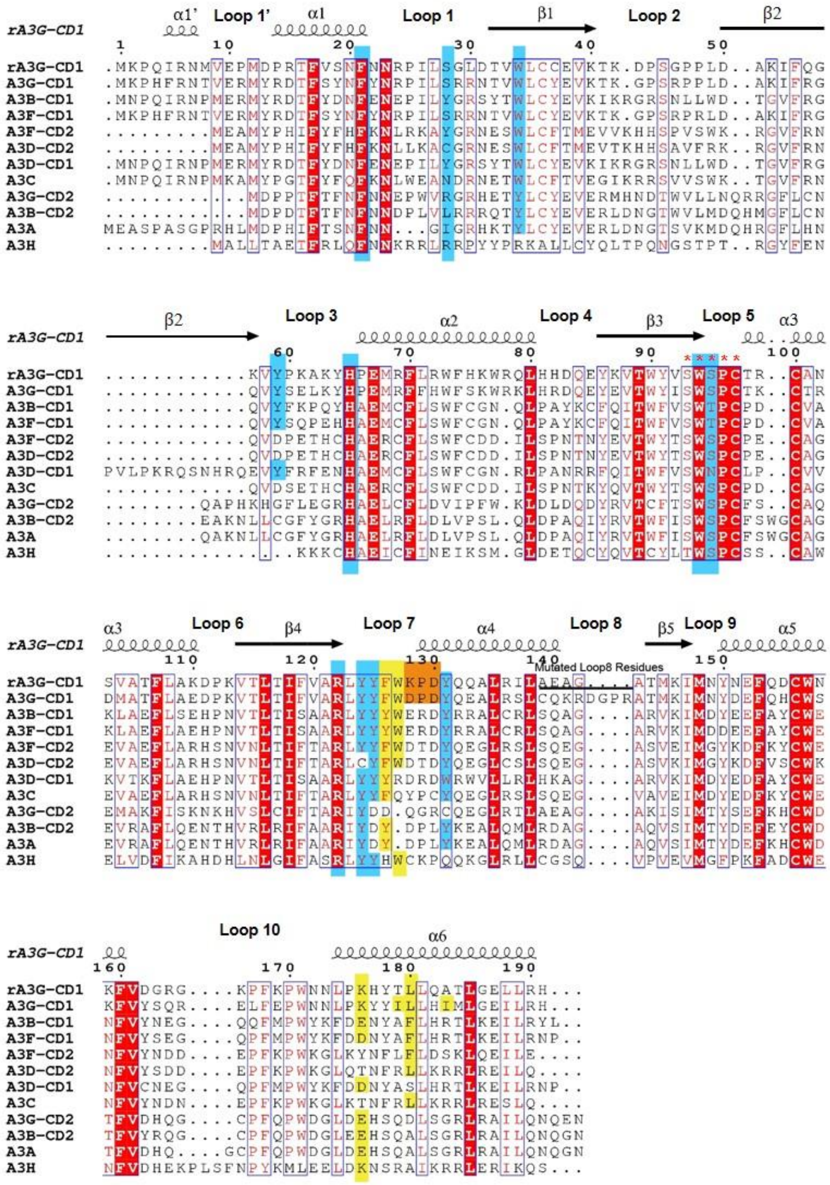

Supplementary Fig. 1. Sequence alignments of rA3G-CD1 (with mutated loop-8) with hA3G-CD1 and other A3s. Numbering of the sequences and depiction of secondary structural elements are based on rA3G-CD1 and are shown above the sequences. Yellow highlighted residues are critically involved in dimerization of rA3G-CD1 and are conserved in CD1 of hA3G and other double-domain A3s. Red highlighted and blue boxed residues are completely or partially conserved across all A3s respectively. The orange highlighted residues (DPD) were previously shown to be important for Vif-mediated degradation of A3G. Blue highlight indicates the residues that are involved in the direct interactions with poly-dT or conformational changes in rA3G-CD1 upon ssDNA binding, which are also conserved in other A3s. Black underline (mutated loop-8 residues) shows the mutations that were made on the loop-8 of rA3G-CD1, which further improved the solubility of rA3G-CD1 protein. Red stars indicate SWSPC on loop-5 that is one of the signature sequence motifs of APOBECs. The sequence alignment graphic was generated by ESPript.

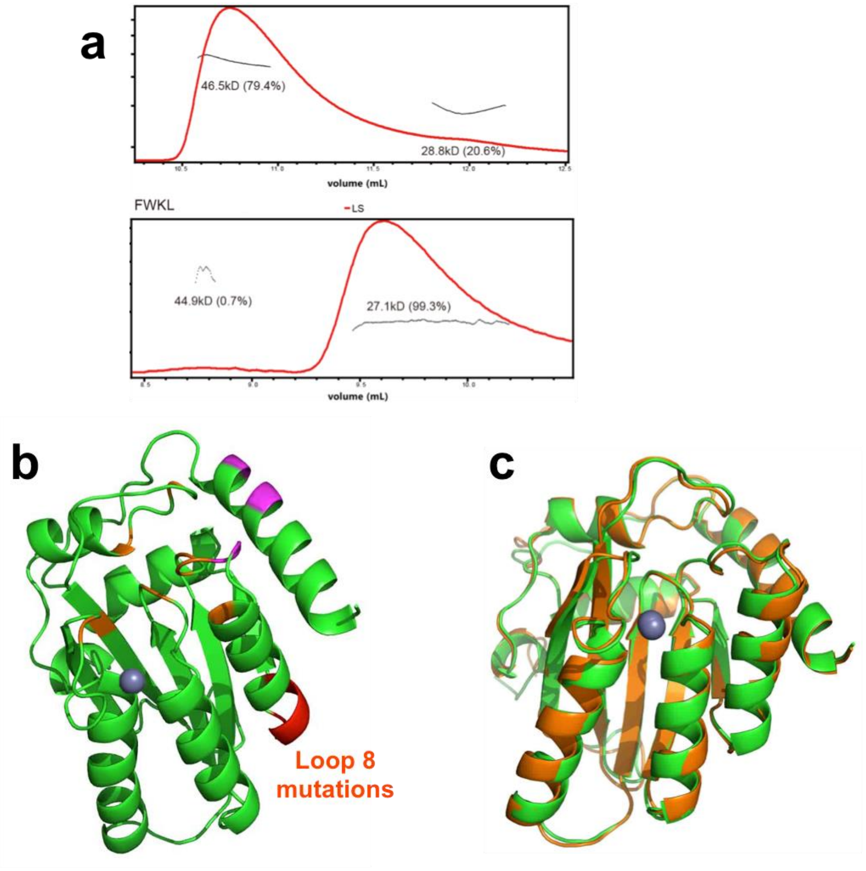

Supplementary Fig. 2. Characterization and crystal structure of rA3G-CD1. (**a**) Multi-angle light scattering (MALS) for rA3G-CD1 (dimer, top panel) and rA3G-CD1 FWKL mutant (monomer, bottom panel). (**b**) Structure of rA3G-CD1, showing the location of the loop-8 mutation at the end of the helix α 4 (red), which is distal from the regions involved in ssDNA binding (orange) and dimerization interface (magenta). (**c**) Superimposition of two apo structures of rA3G-CD1 from two different crystal forms, showing essentially the same structures (including the loops) despite of different crystal packings and different crystallization conditions. Green, CD1-1 (2.0 Å); orange, CD1-2 (2.9 Å).

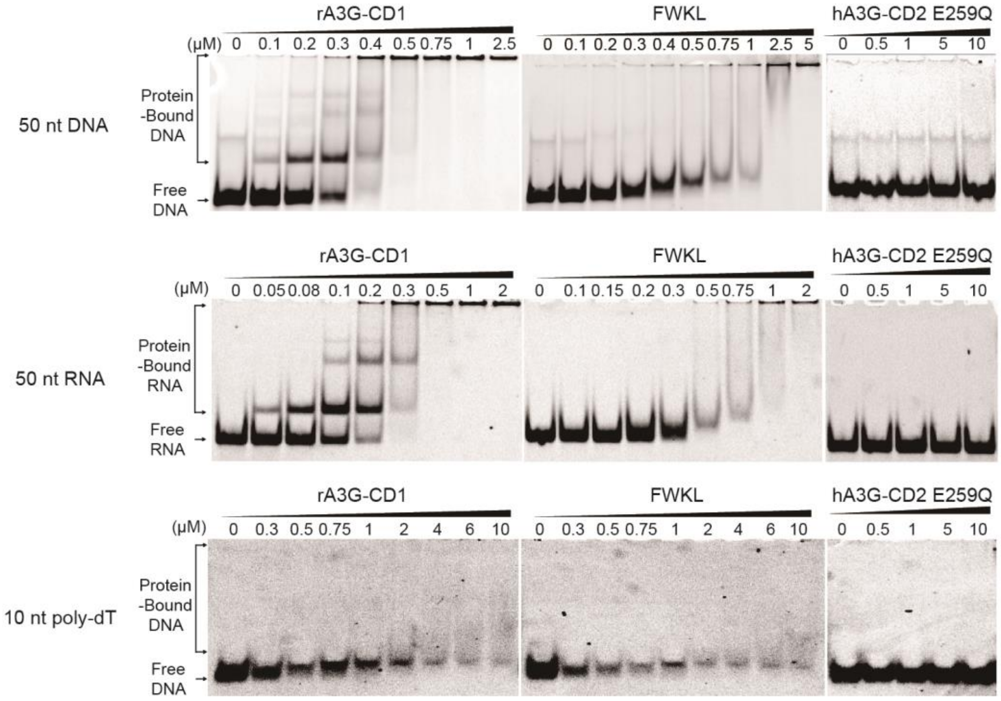

Supplementary Fig. 3. Electrophoresis mobility shift assays (EMSA) of rA3G-CD1, rA3G-CD1 FWKL monomer mutant, and hA3G-CD2 E259Q inactive mutant with various lengths of ssDNA/RNAs, showing the difference in their ability to bind nucleic acids (also see Supplementary Fig. 7 for ITC results). For the gel shift assay with 10 nt poly-dT, the DNA fluorescence signal at the free DNA band position becomes weaker with increasing protein concentration, probably because the highly positively charged rA3G-CD1 in complex with the shorter 10 nt poly-dT may still carry a net positive charge, and thus, may migrate towards the opposite direction and out of the loading wells.

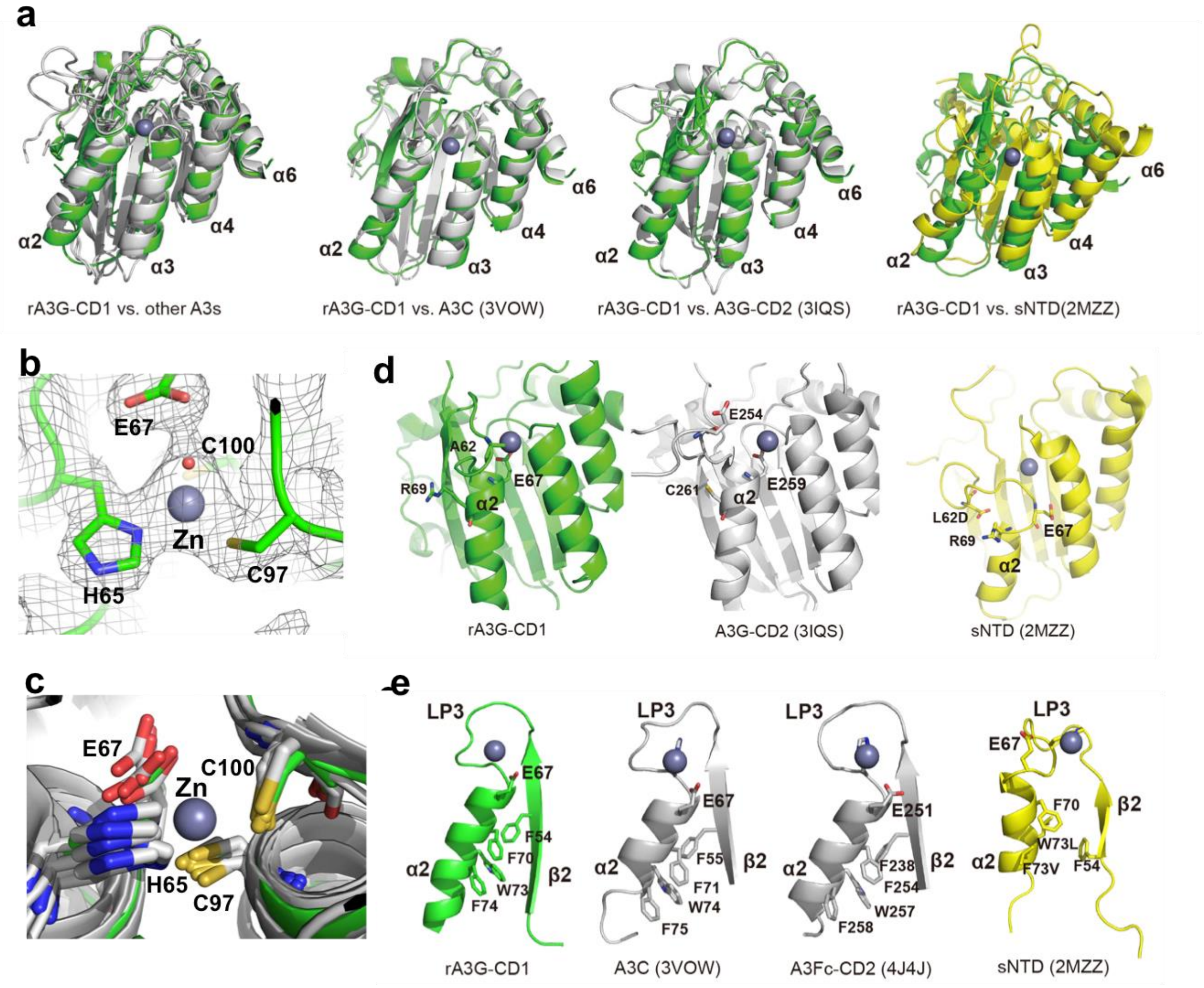

Supplementary Fig. 4. Comparison of the structures of rA3G-CD1 (green), A3G-sNTD (2MZZ, yellow) and other A3s (PDB id: 3IQS/3IR2, 3VOW, 4J4J/4IOU, 5CQH, 4XXO, white). (**a**) The structural superimposition shows that α 2, α 3, α 4 of rA3G-CD1 (green) are aligned well with other A3s (A3A, A3C, A3G-CD2, A3B-CD2, A3F-CD2, left three panels), but not with A3G-sNTD (the right panel). (**b**) The electron density map of rA3G-CD1 structure around the Zn-center (2Fo-Fc, contoured at 1 σ), which are well defined for all residues around the Zn-center. (**c**) The Zn-center conformation aligned very well between rA3G-CD1 and other A3s (A3A, A3C, A3G-CD2, A3B-CD2, A3F-CD2). (**d**) The three side-by-side structures show that the α 2 of rA3G-CD1 is very similar to hA3G-CD2 but different from A3G-sNTD. (**e**) The hydrophobic core structure of α 2-loop3-β 2 is conserved among Z2 structures including rA3G-CD1, A3C and A3Fc-CD2, but not A3G-sNTD. Conserved hydrophobic residues are labeled and shown in sticks. Related loop-3 (panel d) and α 2 (panel e) mutations in A3G-sNTD and their equivalent residues in rA3G-CD1 are also shown in sticks. Here, the L62D mutation in A3G-sNTD may interact with R69 that is exclusively conserved in all CD1s but not in CD2 (panel d). The W73L and F74V mutations in A3G-sNTD appear to alter the core folding of α 2-loop3-β 2.

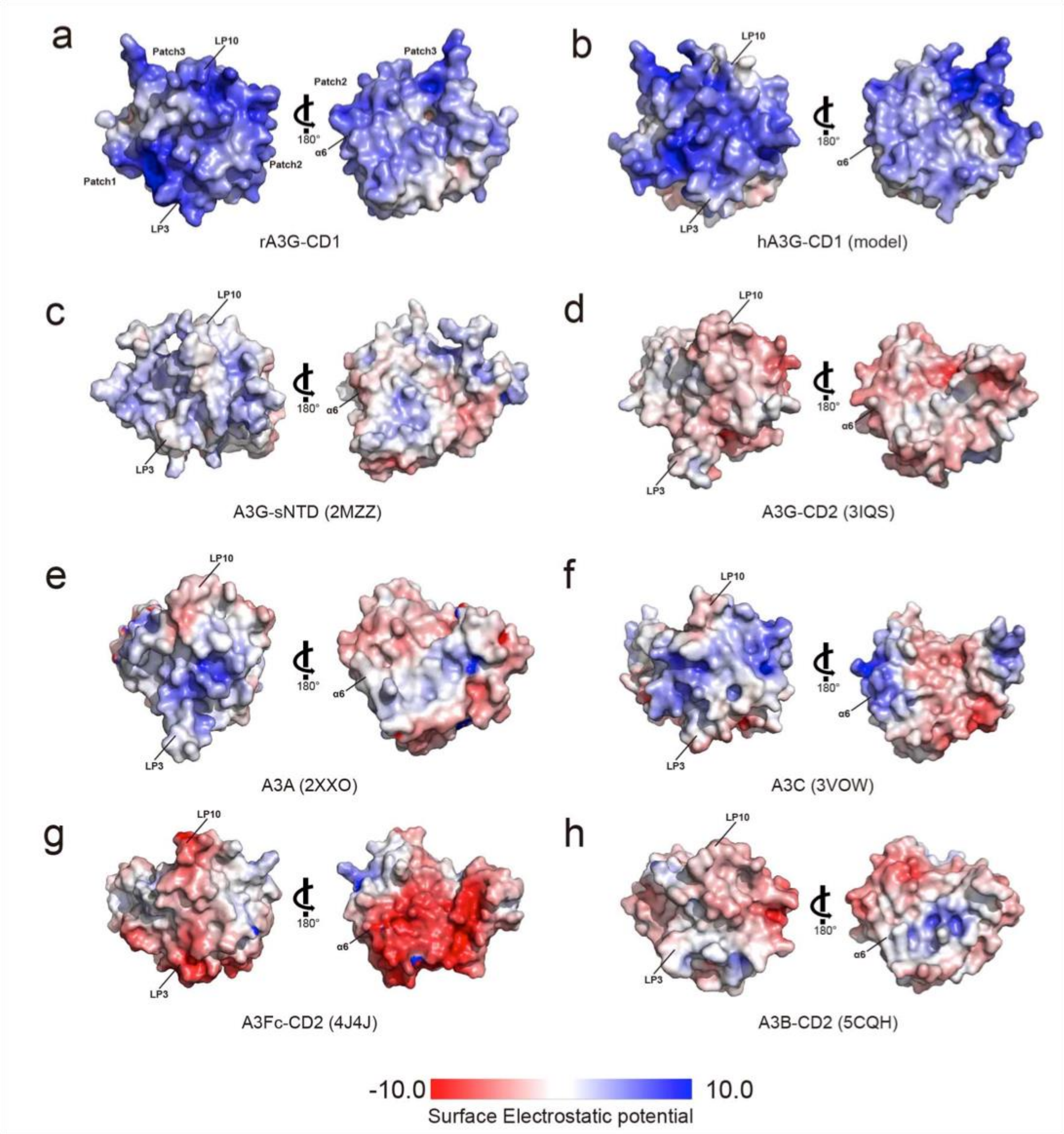

Supplementary Fig. 5. Comparison of the surface electrostatic potential of a rA3G-CD1 monomer with other known A3 structures. Two views related by 180º rotation shown for each structure from panels a-h are all in the same orientations of the deaminase domains of APOBECs. The different shapes of the surface are mostly caused by slightly different structural conformations, loop structures, and amino acid sequences of different A3s. (**a**) rA3G-CD1 crystal structure, showing extensively positively charged surface; (**b**) hA3G-CD1 modeled structure based on rA3G-CD1, showing very similar positively charged surface as rA3G-CD1, but very different from other known A3 structures. (**c**) A3G-sNTD NMR structure (2MZZ), showing very different surface charge characteristics and surface structural features from those of rA3G-CD1 (panel-a) and hA3G-CD1 (panel-b). (**d, e, f, g, h**) Crystal structures of A3G-CD2 (3IQS), A3A (4XXO), A3C (3VOW), A3Fc-CD2 (4J4J) and A3B-CD2 (5CQH) all show much less positively charged surface compared with rA3G-CD1 and hA3G-CD1. The scale of electrostatic potential for all structures is from -10 kT/e (red) to 10 kT/e (blue), which is the same as in Fig. 2e.

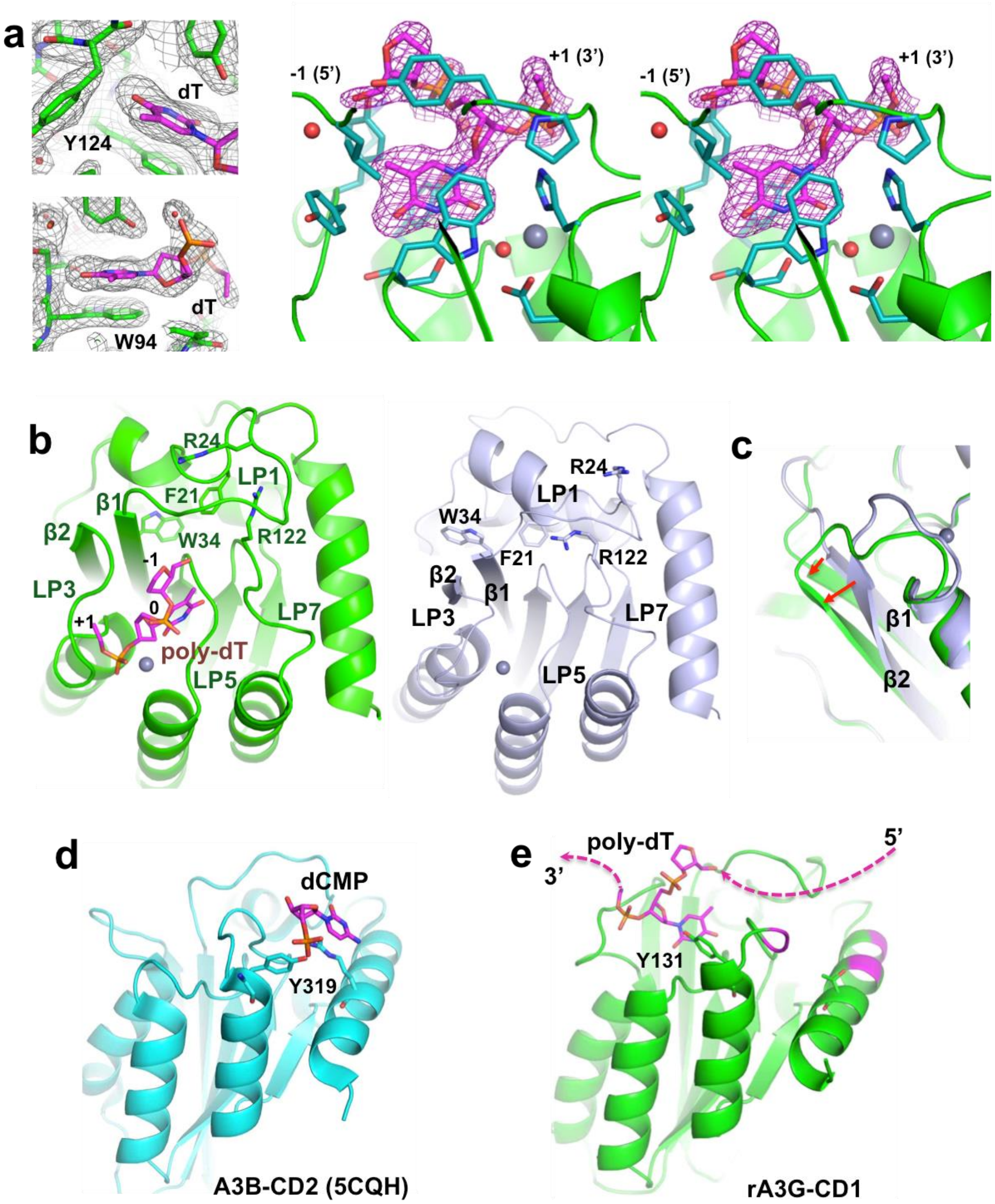

Supplementary Fig. 6. The structure of rA3G-CD1 bound to poly-dT ssDNA. (**a**) **Left panel:** two views of the electron density maps for the dT inserted into the Zn-center pocket and the surrounding amino acids residues. **Right panel:** a stereo-image to show the electron density covering the three nucleotide residues at the Zn-center, with one complete dT residue inserted deep into the Zn-center pocket, the phosphate backbone and a sugar moiety for the residue on the 5' end, and the phosphate backbone for the residue on the 3' end. Density maps are 2Fo-Fc contoured at 1.0 σ level. (**b**) Conformational changes around the Zn-center pocket upon ssDNA binding. The amino acids (F21, R24, W34, R122) in addition to those shown in Figure 3, together with loops 1, 3, 5, 7 (LP1, LP3, LP5, LP7), show conformational changes upon binding to poly-dT. (**c**) A different view shows the conformational β 1 and β 2 shift about 25 degrees upon binding to ssDNA. (**d**) A3B-CD2 in complex with a free nucleotide dCMP (5CQH) bound outside the Zn-center. (**e**) rA3G-CD1 in complex with poly-dT ssDNA, with three nucleotides bound at the Zn-center. The 5' and 3' ends for the three nucleotides of the poly-dT bound to rA3G-CD1 are indicated.

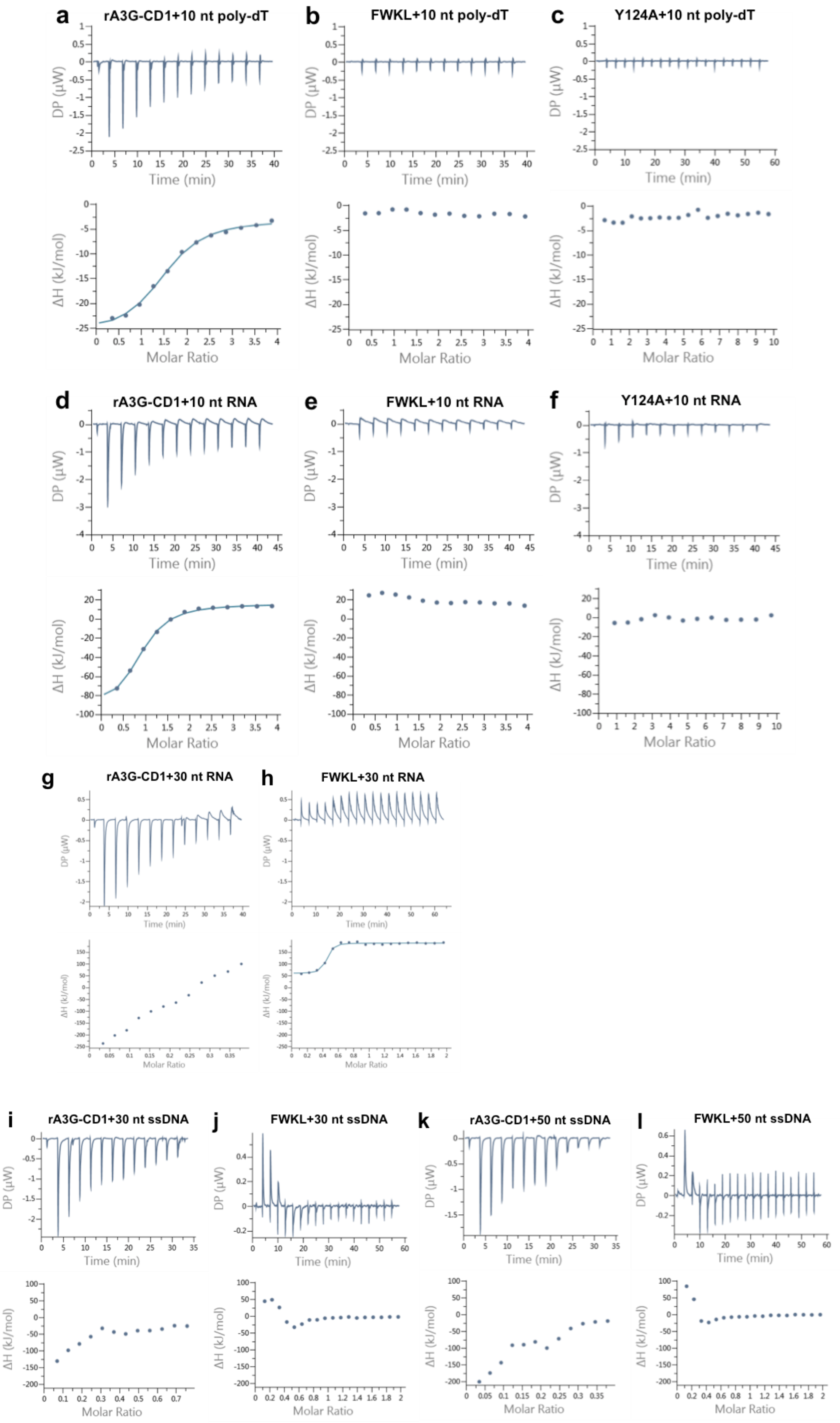

a
rA3G-CD1+10 nt poly-dT
b
FWKL+10 nt poly-dT
c
Y124A+10 nt poly-dT
d
rA3G-CD1+10 nt RNA
e
FWKL+10 nt RNA
f
Y124A+10 nt RNA
g
rA3G-CD1+30 nt RNA
h
FWKL+30 nt RNA
i
rA3G-CD1+30 nt ssDNA
j
FWKL+30 nt ssDNA
k
rA3G-CD1+50 nt ssDNA
l
FWKL+50 nt ssDNA
DP (µW)
Time (min)
ΔH (kJ/mol)
Molar Ratio

Supplementary Fig. 7. Isothermal titration calorimetry (ITC) assays for the binding of rA3G-CD1 and its mutants with 10 nt poly-dT (**a**-**c**), 10 nt RNA (**d**-**f**), 30 nt RNA (**g**, **h**), 30 nt ssDNA (**i**, **j**), and 50 nt ssDNA (**k**, **l**). The ITC data shows that rA3G-CD1 binds to 10 nt ssDNA and RNA, and the FWKL and Y124A mutants had no detectable binding to these oligonucleotides (panels a-f). rA3G-CD1 and the FWKL mutant also showed distinct binding ability to longer oligonucleotides (30 nt and 50 nt). Top parts of each panel show the raw ITC data on heats of binding when oligonucleotides were mixed with the indicated proteins. Bottom parts of each panel show normalized heat of binding based on the ratio of protein and oligonucleotides concentrations. The binding of rA3G-CD1 with 10 nt poly-dT and 10 nt RNA can be fitted into one set of sites binding model and the resulting Kd and entropy were calculated and shown in Supplementary Table 2. The binding of 30 nt or longer oligonucleotides cannot be fitted into typical binding models (**g**-**l**), possibly due to multiple sites of binding on longer ssDNA or protein. All results were representatives from independent repeated experiments.

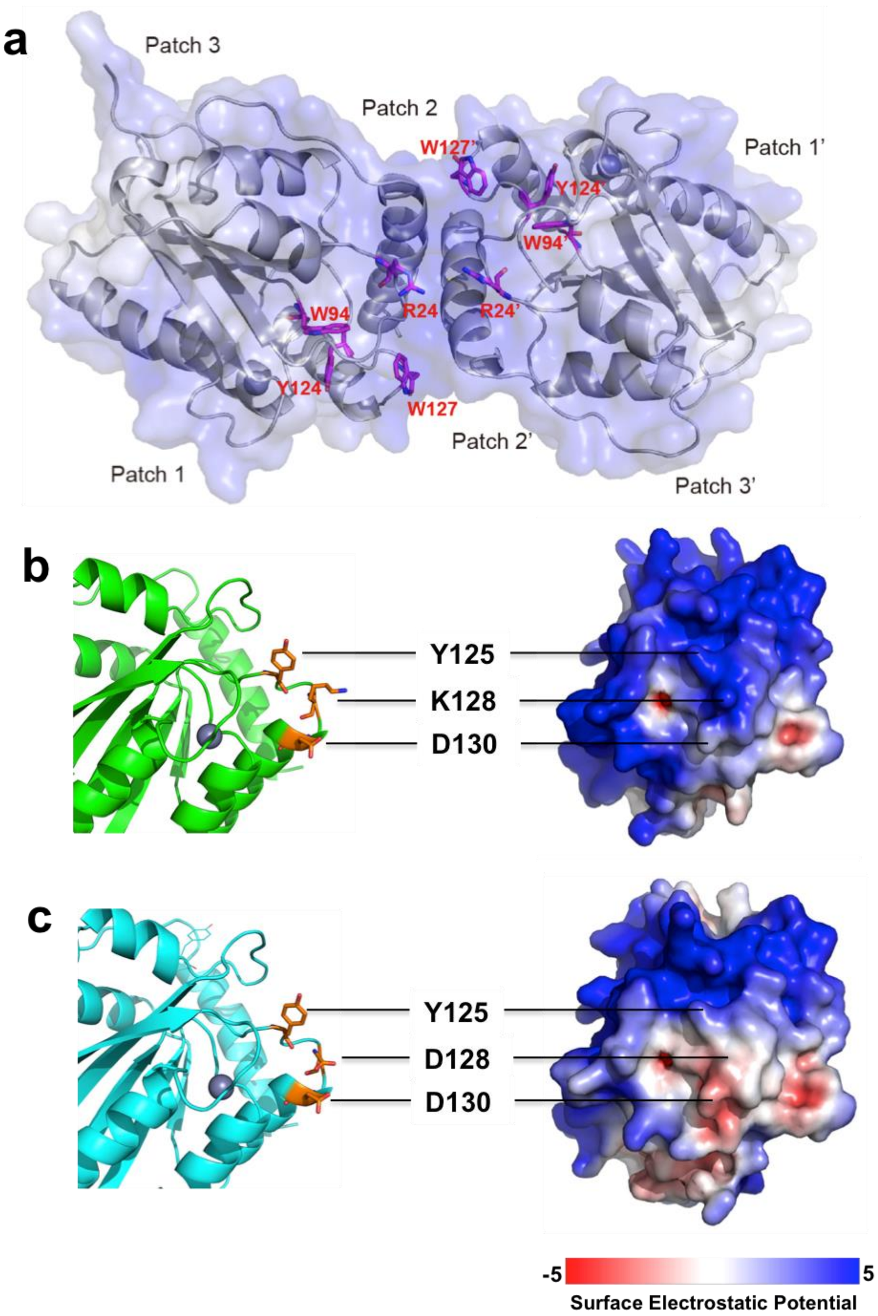

Supplementary Fig. 8. rA3G-CD1 structure provides explanations for the previous reports. (a) Conserved residues reported to be important for RNA binding (R24, W94, Y124 and W127, drawn as sticks) are located along the dimer interface area of rA3G-CD1. (b, c) Interface of rA3G (green, panel-b) and hA3G (cyan, modeled by rA3G-CD1 structure, panel-c) to HIV-1 Vif. Left panels show the structures with the critical residues Y125, D/K128 and D130 colored in orange. Right panels show the accessible surface areas colored according to calculated electrostatic potential from -5 kT/e (red) to 5 kT/e (blue).

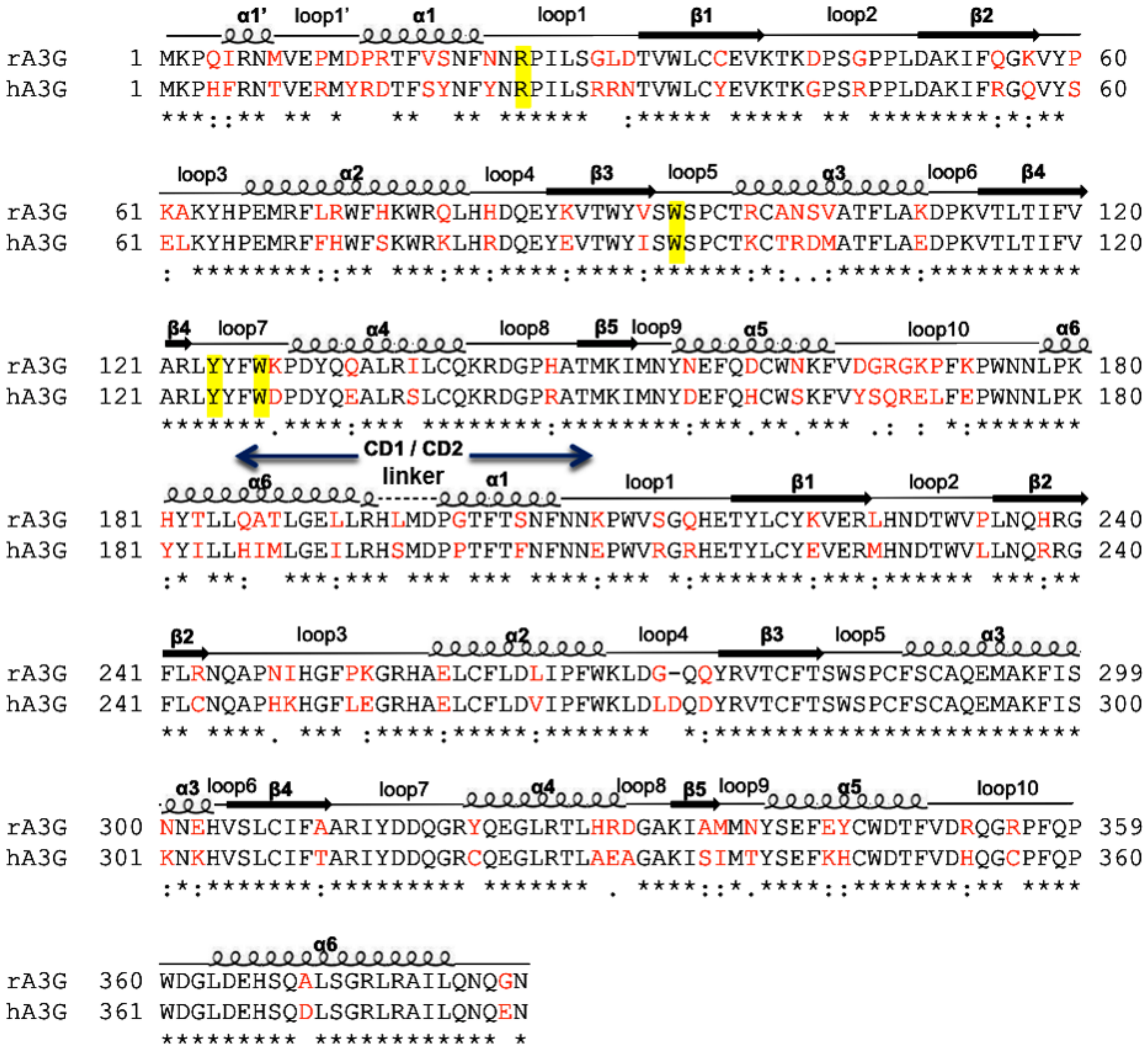

Supplementary Fig. 9. Pairwise sequence alignment between WT rA3G and hA3G. Dash line indicates the linker between CD1 and CD2. Asterisk(*), colon(:) and period (.) indicate different levels of conservation between rA3G and hA3G based on Gonnet PAM 250 matrix. Yellow highlighted conserved residues are previously reported to be important for RNA binding (R24, W94, Y124 and W127).

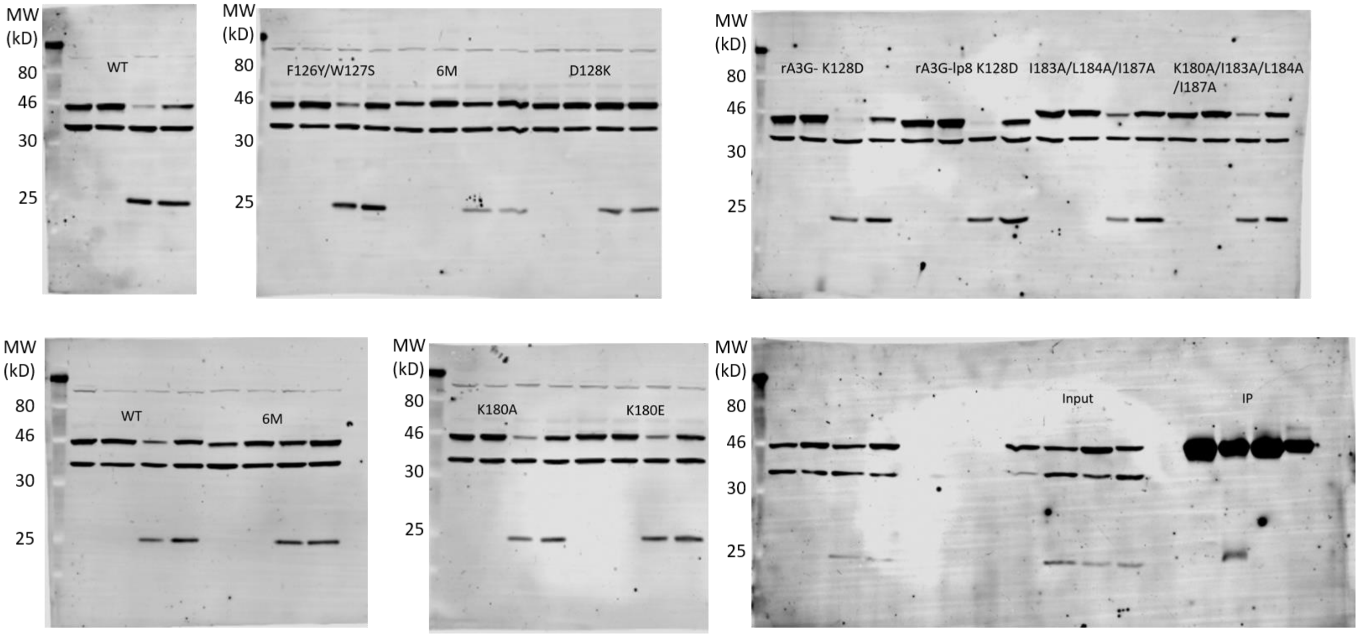

Supplementary Fig. 10. Uncropped scans of western blots of Vif-mediated degradation assay and co-immunoprecepitation shown in Fig. 4a and 4c. The first left lane is the molecular marker.

Supplementary Table 1. The positively-charged residues located at the positively-charged surface patches 1-3.

Patch 1	Patch 2	Patch 3
K52	R24	K2
K57	K128	R6
K61	K180	R15
K63	H181	R168
R69		K170
R72		K173
K76		
R99		

Supplementary Table 2. ssDNA/RNA binding Kd (μM) of rhA3G-CD1 and mutants

Protein	Substrate	Kd (μM)	ΔH (kJ/mol)	-TΔS (kJ/mol)	n
rhA3G-CD1	10 nt dT ssDNA	1.57±0.31	-23.2±1.15	-9.96	1.47±0.04
rhA3G-CD1	10 nt RNA	1.49±0.18	-112±4.17	79.2	0.83±0.02
FWKL mutant	10 nt dT ssDNA	n.d	n.d	n.d	n.d
FWKL mutant	10 nt RNA	n.d	n.d	n.d	n.d
Y124A mutant	10 nt dT ssDNA	n.d	n.d	n.d	n.d
Y124A mutant	10 nt RNA	n.d	n.d	n.d	n.d

n.d., not detected, binding is too weak to be detected.

Supplementary Table 3. Sequences of oligonucleotides used in ITC and EMSA

10 nt ssDNA poly-dT	TTT TTT TTT T
10 nt RNA	UAU UCA UUA U
30 nt RNA	AUU UAU AUU AUU UAU CCC UAU UUA UAU UUA
30 nt ssDNA	ATT TAT ATT ATT TAT CCC TAT TTA TAT TTA
50 nt RNA	AUU AUU AUU AUU UAU CCC UAU UUA UAU UUA UUG UUA UUG UUA UUG UUA UA
50 nt ssDNA	ATT ATT ATT ATT TAT CCC TAT TTA TAT TTA TTG TTA TTG TTA TTG TTA TA
